# Environmental Applications of GM Microorganisms: Tiny Critters Posing Huge Challenges for Risk Assessment and Governance

**DOI:** 10.3390/ijms26073174

**Published:** 2025-03-29

**Authors:** Michael F. Eckerstorfer, Marion Dolezel, Marianne Miklau, Anita Greiter, Andreas Heissenberger, Karen Kastenhofer, Freya Schulz, Kristin Hagen, Mathias Otto, Margret Engelhard

**Affiliations:** 1 Team Landuse & Biosafety Unit, Umweltbundesamt–Environment Agency Austria, Spittelauer Lände 5, 1090 Vienna, Austria; marion.dolezel@umweltbundesamt.at (M.D.); marianne.miklau@umweltbundesamt.at (M.M.); anita.greiter@umweltbundesamt.at (A.G.); andreas.heissenberger@umweltbundesamt.at (A.H.); 2 Institute of Technology Assessment, Austrian Academy of Sciences, Bäckerstraße 13, 1010 Vienna, Austria; karen.kastenhofer@oeaw.ac.at (K.K.); freya.schulz@oeaw.ac.at (F.S.); 3 Division Assessment Synthetic Biology, Enforcement Genetic Engineering Act, Federal Agency for Nature Conservation, Konstantinstrasse 110, 53179 Bonn, Germany; kristin.hagen@bfn.de (K.H.); mathias.otto@bfn.de (M.O.); margret.engelhard@bfn.de (M.E.)

**Keywords:** genetically modified microorganisms, GMM, GM microalgae, GM biofertilizer, biosafety, environmental risk assessment, ERA, monitoring, governance, sustainability assessment, technology assessment, TA

## Abstract

In recent years, the interest in developing genetically modified microorganisms (GMMs), including GMMs developed by genome editing, for use in the environment has significantly increased. However, the scientific knowledge on the ecology of such GMMs is severely limited. There is also little experience at the hands of regulators on how to evaluate the environmental safety of GMMs and on how to assess whether they provide sustainable alternatives to current (agricultural) production systems. This review analyzes two different GMM applications, GM microalgae for biofuel production and nitrogen-fixing GM soil bacteria for use as biofertilizers. We assess the challenges posed by such GMMs for regulatory environmental risk assessment (ERA) against the background of the GMO legislation existing in the European Union (EU). Based on our analysis, we present recommendations for ERA and the monitoring of GMM applications, and in particular for the improvement of the existing EU guidance. We also explore whether existing approaches for technology assessment can provide a framework for the broader assessment of GMM applications. To this end, we recommend developing and implementing an evidence-based sustainability analysis and other methods of technology assessment to support decision making and to address broader societal concerns linked to the use of GMM applications in the environment.

## 1. Introduction

The methods of modern biotechnology, i.e., recombinant DNA technology or genetic engineering, were initially developed from and in microorganisms [1]. Similarly, the most prominent tool for genome editing, CRISPR-Cas technology, was developed from a defense mechanism protecting bacteria against foreign (bacteriophage) DNA [2]. Furthermore other recent developments in biotechnology, such as the convergence of artificial intelligence and synthetic biology, are beginning to be applied to engineering microbes and designing genetic elements [3].

Genetic engineering and genome editing are currently used to develop genetically modified (GM) organisms for release into the environment, e.g., for agricultural applications. GM crop plants, such as GM soybean, maize, cotton, rice, and canola, have been employed in agriculture since the 1990s [4] and regulatory oversight of biotechnology applications was established in the EU and internationally, based on scientific considerations [5]. While such biosafety frameworks typically address all GMOs, the focus is on GM plants and—more recently—on genome-edited plants [6,7] and their risk assessment [8,9,10]. In recent years, some countries have exempted genome-edited plants from the biosafety legislation [11].

In addition to GM plants, GM microorganisms (GMMs) are being developed for use in the environment. In 2015, the OECD identified a variety of potential use scenarios for GMMs [12]. Since then, further studies have been conducted to identify GMM applications in development and/or in (commercial) use, for example, a recent study commissioned by the European Food Safety Authority (EFSA) has explored the applications of different GMMs developed by emerging biotechnological methods in food and feed production [13]. Other activities directed at the horizon scanning of applications of (micro)organisms developed by methods of synthetic biology have been undertaken by the Secretariat of the Convention on Biological Diversity (SCBD) [14]. Another survey of applications for the environmental use of GMMs, including GM microalgae, was published recently by Miklau et al. [15]. This review identified a range of GMMs which were developed for the bioremediation of environmental pollutants, including organic and inorganic pollutants, biocontrol of agricultural and medical pests, including paratransgenesis in pest insects, GMMs as biofertilizers, and GMMs with plant growth promoting effects, as well as GMMs for the production of food, feed, and biofuels as well as other valuable biological substances [15]. Additional overviews on the development of GMMs such as GM entomopathic bacteria and fungi or GM viruses for use in biocontrol of (agricultural) pests have been provided by Azizoglu et al. [16], Bonaterra et al. [17], and Eckerstorfer et al. [18]. Liu et al. [19] reviewed the clinical applications of GM bacteria. The environmental use of GMMs for the removal of inorganic and organic pollutants by bioremediation was reviewed by Pant et al. [20]. Applications of GM microalgae developed for different purposes, including the production of fuels, foods, feeds, and therapeutics, were covered by Sproles et al. [21] and Liang et al. [22], and further applications of GM microalgae are listed in Dolezel et al. [23]. Typically, a mix of different methods for genetic modification, including transgenesis and new genomic techniques, such as genome editing, were used to develop these GMM applications [15,18]

GMMs, particularly GMMs developed by transgenesis, are subject to national biosafety laws in most countries and covered by international treaties such as the Cartagena Protocol on Biosafety (CPB) [24]. Such regulations require the authorization of the environmental release of GMOs and their use in foods and feeds based on a risk assessment conducted for individual applications prior to their approval [25]. The relevance and importance of a framework for a risk assessment of GMMs is stressed by regulators and scientists considering the potential environmental and health risks of such GMM applications [16,18,25]. While considerations concerning the risk assessment of GMMs in regulatory frameworks other than the EU are certainly relevant, an in-depth analysis of the different regulatory frameworks is outside of the scope of this review. Further information on the regulatory status of GMM applications are presented by Hanlon and Sewalt [26]. In addition, recent developments concerning the regulatory oversight of GMMs in the USA were discussed by Shams et al. [27] and Ahmad et al. [28].

This review provides a discussion of the environmental applications of GMMs identified by Miklau et al. [15]. It specifically focuses on the challenges and open questions regarding the environmental risk assessment (ERA) of GMMs in the EU based on relevant examples of GMM applications, such as GM microalgae for biofuel production and GM bacteria developed as biofertilizers. In addition, our review explores how a framework for sustainability analysis and technology assessment (TA) could facilitate a broader assessment of GMM applications. Due to the limited information which is currently available on GMMs, we do not attempt to conduct such comprehensive assessments. Rather, our review aims to identify areas for which further ERA guidance needs to be developed and where additional (biosafety) research is required, e.g., to address the ecological interactions of GMMs in the environment, explore societal opinions and concerns concerning the use of GMMs, and establish fit-for-purpose assessment frameworks.

## 2. Case-Study Examples of Emerging GMM Applications

### 2.1. GM Microalgae for Biofuel Production

One example for the genetic modification of unicellular eukaryotic microorganisms by genome editing that was identified during a recently conducted horizon scanning exercise [15] is the development of GM microalgae with an increased potential for the production of biofuels by metabolic engineering [29,30,31]. The CRISPR-Cas method has been used to modify the lipid biosynthetic pathways in different microalgae, e.g., by inducing overexpression of key catalytic enzymes for fatty acid biosynthesis [30,31]. Engineering the synthesis route of specific fatty acids, such as triglycerides and specifically the storage lipid triacyl-glycerol (TAG), is particularly promising. In order to achieve the increased accumulation of TAGs, the simultaneous overexpression of several key enzymes has been targeted in different microalgal species, as reviewed by Zhu et al. [31]. Genome editing approaches for the modification of the fatty acid synthesis are mostly carried out in model species such as *Nannochloropsis* sp. or *Chlamydomonas* sp. [32]. Since these microalgae were identified in a recent horizon scan as taxa which are the most frequently used for biofuel production [15], they are discussed as case studies in this review.

In the EU, microalgae products are mainly produced for food and feed purposes (e.g., as food supplements), as well as in cosmetic and pharmaceutical products or as fertilizers and biostimulants [33]. The European Commission (EC) has recognized that the cultivation and production of microalgae needs to be up-scaled throughout the EU to meet the targeted goals for production of renewables, e.g., biofuels [34]. This up-scaling, i.e., the high-volume cultivation of GM microalgae, e.g., for biofuels, is likely to take place in open or semi-open ponds (e.g., raceway ponds), being the more cost-efficient production system [35,36] and can be considered a deliberate release into the environment [12]. Production in fully closed systems (either indoor or outdoor) is a contained use subject to Directive 2009/41/EC in the EU.

#### Case Study 1: GM *Nannochloropsis gaditana* and GM *Chlamydomonas reinhardtii* for the Production of Biofuels

The genus *Nannochloropsis*, unicellular algae belonging to the division of Ochrophyta, is particularly relevant for biofuel production due to its naturally high lipid content (mostly saturated long-chain fatty acids), high growth rates, and its ability to maximize lipid production, including TAGs, under nitrogen starvation [32]. Different *Nannochloropsis* species occur mainly in marine environments, tolerating a wide range of environmental variations at least under laboratory conditions [36]. Some strains of *N. oceanica* also grow in freshwater and the freshwater species *N. limnetica* may have evolved from marine relatives [37,38]. Due to the lack of morphological differences, individual species can only be distinguished by analyzing genetic data, e.g., by genome sequencing. In addition, knowledge on the life cycle of some species, including *N. gaditana*, under natural conditions is largely lacking. Genetic transformation methods were successfully developed a decade ago [39] and genome editing methods, particularly CRISPR-Cas, have been applied in these two taxa starting from 2016 [40]. However, some genome editing approaches were found to be too inefficient [36].

In a proof-of-concept study, Ajjawi et al. [41] identified a genetic switch that regulates the conversion of carbon to lipids in *N. gaditana*. In their work, the authors used CRISPR-Cas9 and a RNAi mechanism to attenuate the expression of a transcription factor for lipid accumulation, Zn(ii)2Cys6 (ZnCys). The modification resulted in the decrease in ZnCys mRNA and increased carbon partitioning to lipids. All GM lines showed increases of up to 100% in the production of fatty acid methyl esters, as well as a decreased protein content. Polyunsaturated fatty acids (C20:5) decreased from 23 to 6% due to an upregulation of desaturases and elongases. The C:N ratios increased to a maximum of 25 in modified algae as compared to 7 in the wild-type algae. Thus, a high biomass productivity was achieved under N-limiting conditions indicating that the GM trait changed the allocation of carbon into TAGs [41]. A similar enhancement of lipid productivity in the same algal taxon by attenuation of ZnCys expression with a different CRISPR-Cas based system was achieved by Verruto et al. [42].

*Chlamydomonas* sp. are flagellated green algae belonging to the Chlorophyta division. *C. reinhardtii* is considered a suitable species for the application of genome editing approaches due to its well-known genetics and the availability of the nuclear, chloroplast, and mitochondrial genome [22]. *C. reinhardtii* is primarily a soil-dwelling species, but has also been reported from freshwater lakes [43]. Therefore, the distribution of *C. reinhardtii* in the environment as well as its life-history is not well understood leaving knowledge gaps with respect to the function, abundance, growth, and life cycle of natural populations [43,44]. As a model species, *C. reinhardtii* has been compared to yeast and termed the “photosynthetic” or “green” yeast [45]. *C. reinhardtii* is known to have a highly polymorphic genome and thus is able to efficiently adapt to its environment [46]. As reviewed by Ghribi et al. [47], genome editing in this microalgae is challenging, due to a low efficiency of editing when using standard CRISPR-Cas9 systems, significant off-target activity of these editors, and a high degree of instability of the introduced genetic modifications. Kao and Ng [48] used CRISPRi, a method for the targeted silencing of transcription [49], in *C. reinhardtii* to increase biomass and lipid productivity by silencing the *pepc1* gene (coding for phosphoenolpyruvate carboxylase isoform 1). Silencing of the *pepc1* gene affects the carbon flux in the biosynthesis pathway of triacylglycerols in the modified microalgae, which significantly increases lipid content compared to the wild type [50].

For the two examples, little is known about the overall biochemical changes in addition to those targeted by the genetic modification. The transcription factor Zn(II)2Cys6 is a homolog of the *cys6* gene family in fungi. Cys6 zinc cluster proteins are ubiquitous transcriptional regulators for different biological processes such as carbon or nitrogen utilization, amino acid metabolism, or sexual development, but are also involved in stress response or secondary metabolite production (see discussion in Zhang et al. [51]). Due to the unspecific role of the targeted transcription factor in algal cells, further metabolic and phenotypic traits may be affected. Ajjawi et al. [41] analyzed the gene regulation in the modified microalgal lines and found that the significantly upregulated genes were mainly relevant for protein synthesis, while significantly downregulated genes, such as *pepc*, were mostly involved in photosynthesis and light harvesting in *C. reinhardtii*. *pepc* genes are present in various plants, archaea, bacteria, and microalgae [48] and play an important role in photosynthetic CO_2_ assimilation in these organisms [52].

### 2.2. GM Bacteria for Use as Biofertilizers

Some of the studies identified during the horizon scan for GMM applications conducted by Miklau et al. [15] addressed GM biofertilizers, i.e., GMMs that produce and excrete bioavailable nitrogen and thereby promote the growth of crop plants [53,54]. These studies focused on improvements of *Azotobacter vinelandii* by genetic modification to increase their biofertilizing capacity. GM approaches in different species of free-living, plant-associated soil bacteria, namely *Klebsiella variicola*, include genetic modifications introduced by genome editing [55,56].

A number of biofertilizer products containing nitrogen-fixing bacteria are commercially used in Asian countries, e.g., China, and also in Canada [57]. Prior to the easy availability of synthetic fertilizers, biofertilizers based on free-living, diazotrophic bacteria have also been commonly used for more than a century in Europe, particularly in the former USSR and eastern European countries, to support the cultivation of different cereals, oilseed plants, e.g., mustard and sunflower, and various vegetables including sugar beet, carrot, and potato [54]. Several companies, e.g., the US company Pivot Bio, are developing GM biofertilizers to increase maize productivity, such as GM *K. variicola* strain Kv137-1036, commercially available in the USA since 2019 [56] and recently in Brazil [55].

#### Case Study 2: GM *Klebsiella variicola* and GM *Azotobacter vinelandii* as Biofertilizing Agents

The applications in *Azotobacter vinelandii* and *Klebsiella variicola*, reported in Das [54] and Wen et al. [56], focused on the modification of the *nif*-genes in these diazotrophic bacteria, i.e., bacteria which are able to convert atmospheric nitrogen into bioavailable nitrogen compounds such as ammonium. The *nif* genes are responsible for the natural ability of the respective bacteria to fix atmospheric nitrogen into nitrogen-compounds such as ammonia, which are bioavailable for crop plants. However, the expression of the *nif* genes, which are relevant for the biological nitrogen fixation process, is usually tightly regulated in naturally occurring diazotrophic bacteria. The expression of these genes is repressed by nitrogen-containing substances such as ammonia or urea. The genetic modifications in the developed GMMs decouple the regulation of nitrogen fixation from the occurrence of exogenous nitrogen compounds by deleting a regulatory gene (*nifL*), which represses the bacterial nitrogenase genes when exogenous nitrogen is present in the environment. Additionally, an activator of nitrogenase expression (*nifA*) is placed under the control of a constitutive promoter [54,56,58]. These genetic modifications result in the increased expression of the genes, which form the bacterial nitrogenase enzyme (NifHDK). In some studies, additional copies of the *nifH* gene were inserted to further increase the amount of nitrogenase in the resulting GM *A. vinelandii* strains [54] and genes coding for ammonium transporter proteins were modified to maximize ammonium excretion [59]. Additionally, the effects of environmental factors, such as increased molybdenium levels, leading to higher ammonium excretion were determined [59] However, it has also been shown that mutations resulting in the silencing of *nifL* expression are not very stable and over time, revertant sub-populations with a higher growth rate, but a lower nitrogen fixing ability will emerge [58].

The *nif* genes expressing the nitrogenase enzyme have also been transferred to other microorganisms, such as *Escherichia coli* and *Saccharomyces cerevisiae*, to introduce a nitrogen fixation ability into non-diazotrophic microbes [60]. Other approaches described by Ambrosio and Curatti [53] have additionally modified the expression of the bacterial glutamine synthetase to increase ammonium excretion by the respective GM *A. vinelandii* strain. A recent overview on GMMs created by synthetic biology approaches [14] identified studies directed to transfer nitrogenase components from *Azotobacter* and *Klebsiella* into endophytic soil bacteria, e.g., *Azorhizobium caulinodans* and *Rhizobium* sp. [61].

The case study at hand focuses on GM *Azotobacter vinelandii* and *Klebsiella variicola* intended to support the cultivation of cereal crops. Non-modified strains of diazotrophic bacteria have been used as biofertilizing agents since the early 1900s [54]. However, due to the tight control of the nitrogen-fixation pathway in non-GM diazotrophic bacteria, only GM bacteria are able to fix the relevant levels of bioavailable nitrogen in the relatively nitrogen-rich soils which are currently used for cereal production [56]. To achieve the targeted disruption of expression of the nif-cluster genes in *Azotobacter vinelandii* and *Klebsiella variicola*, CRISPR-Cas based systems for genetic modification were developed for both bacteria. These systems either facilitate the total knockout of expression of the respective target genes [56] or the transient knockdown of expression of target genes [62].

The genus *Azotobacter* was first described in 1901 and belongs to a group of Gram-negative soil bacteria (Gammaproteobacteria), which is common in soils of all parts of the world [54]. *Azotobacter* cells, including *A. vinelandii*, are found in soil environments in the rhizosphere of plants and they are also present in different water bodies. In aquatic environments, gammaproteobacteria are associated with microalgae in a microenvironment called the phycosphere, which plays a major role in the ecosystem productivity and in nutrient cycling [63].

Under conditions which are facilitating propagation and growth, *Azotobacter* is able to fix atmospheric nitrogen without association or symbiosis with plants [64]. Inoculation of seeds with *Azotobacter* leads to better yields for different cereal crop species as well as other crop plants [54]. The effect is due to the nitrogen-fixing capacity of the used *Azotobacter* strains and additional plant growth-promoting effects exerted by these bacteria. *Azotobacter* cells excrete a number of plant hormones such as auxins (indole acetic acid, IAA), gibberellin-like substances, and cytokinins. They also provide solubilized phosphate-stimulating plant growth [54]. Furthermore, they suppress phytopathogens, such as bacterial and fungal pathogens (e.g., *Sclerotium* sp., *Fusarium* sp., *Cephalosporium maydis*, *Alternaria brassicola*, and *Colletotrichum falcatum*) and inhibit the early development of plant pests, e.g., nematodes and lepidoptera larvae [65,66].

*K. variicola* is a Gram-negative, non-motile bacterium belonging to the *Klebsiella pneumoniae* complex [67]. It is found in low abundance in the rhizosphere of different plants in a variety of soil ecosystems around the world. In cooperation with other soil bacteria, *K. variicola* supports plant growth directly and indirectly in different ways, e.g., through providing relevant nutrients via biological nitrogen fixation and by solubilizing minerals such as phosphate as well as by promoting plant growth by excreting IAA and other phytohormones [67,68]. Additionally, *K. variicola* suppresses the development of phytopathogens in the soil [67]. However, some strains of *K. variicola* may also colonize plants as an endophyte and together with other endophytic bacteria may cause plant diseases such as banana sheath rot [69]. *K. variicola* is not restricted to terrestrial soil ecosystems, but is also found in aquatic compartments such as rivers and wastewater together with other bacteria [67]. In aquatic environments, different gammaproteobacteria (the class of bacteria to which *Klebsiella* sp. belongs) are frequently associated with microalgae and are components of their phycosphere. Their relationship is important for the productivity and stability of these aquatic habitats and food webs through mutually beneficial interactions [58,63]. The use of consortia consisting of microalgae and N-fixing microorganisms has recently been proposed for different biotechnological applications, e.g., to enhance lipid and biomass productivity, for hydrogen production, and as biofertilizer for crops [58].

## 3. Considerations for the Environmental Risk Assessment (ERA) for GMMs

### 3.1. Overall Framework for an ERA of GMMs in the European Union

In the EU, Directive 2001/18/EC on the deliberate release of GMOs into the environment and the placing on the market of GMOs provides the legislative framework for the regulation and risk assessment of GMM applications for release into the environment, including GMMs developed by new genomic techniques, such as genome editing using CRISPR-based technologies. According to the requirements stipulated in Directive 2001/18/EC, GMMs need to be risk assessed prior to authorization. In addition to the required ERA, GMMs are also subject to mandatory post-market environmental monitoring (PMEM), labelling, and renewal of authorization after 10 years. With regard to the use of new genomic techniques, the limitations of the existing regulations have been discussed by Agapito-Tenfen et al. [70] and in our previous work relating to GM plants [9,71].

The ERA approach for GMMs according to Directive 2001/18/EC is structured in a similar way as the ERA for GM plants [72]. It is conducted in a six-step approach, starting with a problem formulation to identify hazards and exposure pathways associated with the use of a particular GMM (see Dir 2001/18/EC, Annex II, C.2). Ideally, this problem formulation links the potential adverse effects of a GMO to assessment endpoints derived from protection goals in order to derive risk hypotheses, which eventually can be tested for risk characterization during ERA [73]. Annex II of Directive 2001/18/EC also outlines relevant areas of risk in Section D1, which were considered in our analysis of GM microalgae and GM bacteria used as biofertilizer in Section 3.4 and Section 3.5, respectively.

While the general approach to ERA is similar for GMMs developed by transgenesis as compared with GMMs developed by genome editing, a number of specific aspects due to the specific technique applied need to be considered during the assessment. For example, effects resulting from the random integration of transgenic constructs or the random integration of DNA from (plasmid) vector backbones are not relevant for GMMs developed by targeted genome editing methods if no insertion of foreign DNA sequences occurs. In contrast, GMMs developed by genome editing need to be assessed with regard to the absence of constructs for the expression of, e.g., CRISPR-tools if such constructs were previously integrated in the genome of the parental microorganism. During the molecular characterization of genome-edited GMMs, unintended modifications due to the off-target activity of the used genome editing tools have to be considered [74,75].

In 2011, the European Food Safety Authority (EFSA) provided specific guidance for the ERA of GMMs (e.g., [76]) with the focus on GMM applications used for food and feed purposes. This existing guidance for transgenic and genome-edited GMMs was recently reviewed by EFSA on behalf of the EC [77,78]. In December 2024, a draft guidance document for the characterization and risk assessment of microorganisms, including GMMs, in the food chain was published for public consultation [79] and is in revision since February 2025 by EFSA.

A number of different applications of GMMs for environmental use have already been assessed by the respective regulatory agencies in other countries than the EU, including the USA, Brazil, and Canada [15]. Environmental releases of GM microalgae were also reviewed in Australia by the Office of the Gene Technology Regulator [80]. The available information from these assessments is considered for the discussion provided below.

### 3.2. Protection Goals to Be Considered During ERA in the European Union

The approach for the ERA of GMOs as implemented in the EU is devised with a view to the protection goals laid out by the EU legislation or the respective national legislation [81]. The EFSA has published guidance concerning specific protection goals for ERA, including protection goals relating to biodiversity and ecosystem services provided in the receiving environments which are exposed to a GMO [82]. Table 1 provides examples of EU environmental protection goals, which are particularly relevant for the analyzed GMMs. These protection goals are further discussed in the Supplementary Materials (S1).

### 3.3. Exposure of the Environment Through the Intended and Unintended Release of GMMs

Exposure of the environment to GMMs can occur through either intended or unintended releases. The use of biofertilizers containing GM bacteria is based on the intentional release of the GMMs into agricultural soils. However, the intended soil treatment with the GMM is likely to result in secondary, unintended exposure of other ecosystems via the transfer of soil by wind or run-off caused by rain. Agricultural land and water bodies are interconnected in many ways and exchange materials including organic matter and soil [83]. As discussed below, the unintended release of GM microalgae from high-volume, semi-enclosed production facilities is hardly avoidable. Thus, both case studies represent different environmental exposure pathways, which need to be considered during the ERA of GMMs according to the EFSA guidance.

#### 3.3.1. Exposure of the Environment to GM Microalgae During Production of Biofuels

During outdoor cultivation, the exposure of the environment to aerosols containing GM microalgae is unavoidable due to the necessary mechanical mixture of the algal suspension. This can cause dispersal of GM microalgae over large distances, depending on temperature and humidity [84]. Dispersal by air from open pond facilities has been experimentally shown for GM and non-GM microalgae over a distance of at least 150 m [85,86,87]. *Chlamydomonas* sp. has even been identified in the air 1100 m above the ground which can facilitate long-range dispersal over extended ranges [43]. Dispersal of microalgae is also facilitated by animals or human activities, e.g., by birds, insects, or by ballast water [35,38]. The large-scale and long-distance dispersal of microbial eukaryotes is considered the reason for their global distribution [88]. Their small size (e.g., 10 μm for *Chlamydomonas* and 2–5 μm for *Nannochloropsis*) enables dispersal into natural habitats, via different pathways along the production chain, such as cultivation, harvest, processing, disposal, or use. Thus, exposure of the environment to GM microalgae may therefore occur through:

- Dispersal by air in aerosols, or by wildlife, humans or equipment (e.g., if cultivation ponds are not tightly covered);
- Spillage during handling of algae suspensions during production, harvesting, or transport (e.g., via wastewater, drainage water);
- Spillage of microalgae cultures, e.g., due to extreme weather events or floods;
- Failure of containment (e.g., accidental leakage from bioreactors or cultivation ponds).

For experimental open pond trials with GM microalgae, regulatory authorities have demanded the limitation of environmental exposure by requiring physical containment measures to prevent escape and exclude wildlife [38,80,87]. Such containment can be provided by earthen perimeter structures, netting, or coverage of ponds. Some authors have also considered genetic biocontainment measures that reduce the fitness of GM microalgae or their offspring under natural conditions to limit the survival of GM microalgae outside production systems [89,90,91,92]. However, it is uncertain whether such biocontainment approaches work sufficiently well. Biocontainment approaches are based on genetic modifications to prevent the replication or transmission of engineered plasmids, or modifications inducing auxotrophy or dependance on non-standard amino acids or nutrients [25,89,93]. Such mechanisms could also pose additional risks to the environment, e.g., if such GMMs are unintentionally spread to environments which enable their growth [94].

Many microalgae species are able to survive adverse environmental conditions over extended periods due to an ability to form resting stages with reduced metabolism or other survival structures, such as zygospores, which are highly resistant to adverse environmental conditions, including desiccation and freezing [95]. However, the ability to form resting stages is dependent on the respective species, and may last for either short or longer periods (see Sundqvist et al. [96] and references therein). *Chlamydomonas* species may react to adverse environmental stress (e.g., light limitation or nutrient shortage) by the formation of cell aggregates, dormant zygospores, palmelloids, or cysts [44]. *Nannochloropsis* sp. can survive suboptimal conditions, but it is not fully clear whether it can form cysts or specific survival structures [38]. The ability to survive adverse conditions in microalgae could enable their survival and persistence in a range of different environments. Under extreme environmental conditions, which promote the fusion of lipid membranes, *Chlamydomonas* cells may also incorporate certain diazotrophic bacteria, such as *Azotobacter*, which normally do not show an endosymbiotic association with microalgae [58]. The incorporated bacteria are maintained at a level of 1–8 cells/microalgae cell in organelle-like vesicles in the cytoplasm of the microalgae and support the growth of the microalgae in N-deficient medium for extended periods [58]. This highlights the enormous plasticity of the interactions of different microorganisms in the environment, ranging from a mutually beneficial association of independent cells to intracellular symbiosis.

#### 3.3.2. Exposure of the Environment to GM Bacteria Used as Biofertilizers

Environmental exposure to GMM biofertilizers occurs primarily through the treatment of agricultural soils during planting or through the treatment of planting materials with the GMMs by dipping plant seeds or seedling roots in suspensions of GMMs prior to planting [54,56]. The direct application of GM biofertilizer suspensions is described by Wen et al. [56]. Freeze-dried microbial powder containing the *K. variicola* strain Kv137-1036 was inoculated into batches of sterile growth medium and allowed to grow for 2 days prior to the in-furrow application at crop planting. During planting, small amounts of the bacterial suspension were dispensed into the soil close to the crop seeds. With both methods, significant amounts (i.e., approx. 10 million cells/plant or 10 million cells/m^2^) of viable GMMs were released on the treated plots. The released free-living GMMs were intended to colonize the rhizosphere of the soil. Some soil bacteria such as *Klebsiella* or *Kosakonia* live in association with the growing plants, i.e., attached to root surfaces [56,97].

The further spread of the released GMMs from the treated field sites may take place through the movement of inoculated plant material or treated soil material as well as via transport of the GMM by water and to a lesser degree by wind. Another route of exposure is by the accidental release of pre-cultured material, e.g., from the containers holding reconstituted microbial formulations or the formation of aerosols during application. However, the most significant exposure routes will be the intentional treatment of field plots and the subsequent exposure of different water bodies via run-offs after rainfall. Both *Azotobacter* and *Klebsiella* have been isolated from freshwater bodies in all continents and climates, including contaminated rivers and wastewater [54,67]. *Azotobacter* and *Klebsiella* do not produce endospores as resting stages, but secrete outer layers of slime to protect the bacterial cells [54,98]. Under unfavorable environmental conditions, *Azotobacters* form thick-walled cysts, which are able to survive for up to 10 years [54].

The exposure of different environments to significant amounts of GMMs may result in the formation of new microbial consortia, e.g., consisting of (diazotrophic) bacteria, microalgae, and fungi [58] or change the composition of existing microbial communities. As discussed below, this may substantially alter the metabolic performance of the GMMs and the associated microorganisms [99,100], as well as their growth and survival [58,63].

### 3.4. Relevant Risk Issues for the ERA of GM Microalgae Used for Biofuel Production

Until the present, only a few controlled releases of GM microalgae into the environment have been conducted and thus the available experience with risk assessment and risk management with GM microalgae strains is limited. Furthermore, many of the parental microalgae species are not considered domesticated [91]. Australia has approved the limited release of a GM strain of the marine species *Nannochloropsis oceanica*, expressing increased levels of fatty acids. In covered production facilities, the GM algae were tested until 2023 [80]. The risk assessment focused on risks associated with the modified trait. These included the following: (1) the potential toxicity of the increased levels of some fatty acids for humans, and (2) the potentially reduced abundance of desirable water organisms in the biotic environment due to the increased production of certain fatty acids [80]. Due to the toxicity of one of the fatty acids, some predators may reject GM microalgae, thereby increasing their survivability. However, the applicant did not provide data on the palatability of the GM microalgae. In addition, potential effects regarding the uptake of environmental pollutants by microalgae were discussed, and further data to address these remaining uncertainties were considered necessary [80]. For GM microalgae, the environmental risk issues outlined in Table 2 need to be considered.

**Figure 1:**
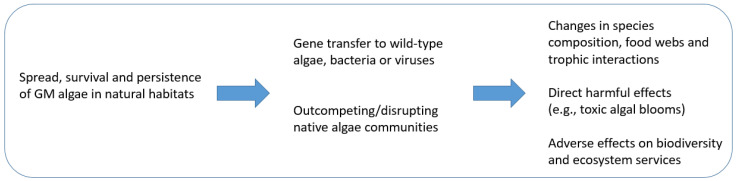
Possible pathways to harm resulting from unintended or unavoidable exposure of natural habitats with GM microalgae used for biofuel production.

The ERA according to Directive 2001/18/EC Annex IIIA (information requirements for other than higher plants) needs to consider the specific biological characteristics of microalgae, e.g., their different feeding mode, including photo-autotrophy [77], and their ability for gene transfer by sexual reproduction. Further, microalgae have specific growth requirements and occupy specific niches, which are often not well characterized [12,101]. With a view to the pathways of exposure and the resulting pathways for harm as shown in Figure 1, the possible adverse environmental effects of GM microalgae are discussed in the following subsections.

The possible pathways to harm resulting from the unintended or unavoidable exposure of the environment to GM microalgae for biofuel production are depicted in Figure 1. Possible adverse effects, which may result from the spillage of GM microalgae from production facilities and their spread, survival, and persistence in exposed natural habitats are further discussed in the following subsections.

#### 3.4.1. Fitness, Survival, and Persistence of GM Microalgae in Natural Habitats

To exert adverse effects in ecosystems and on biodiversity, GM microalgae which are unintentionally released from biofuel production facilities must survive, proliferate, and persist in the exposed natural habitats. The survival and persistence of GM microalgae in such habitats depends on a range of factors, such as the habitat requirements of the respective species, unintended phenotypic traits of the GM microalgae, and the fitness effects of the GM trait under the respective environmental conditions. In addition, the occurrence of other microorganisms, such as bacteria or fungi, may influence the growth and survival of GM microalgae [58].

Szyjka et al. [87] carried out a field trial showing that GM microalgae with increased levels of fatty acid synthesis were able to disperse and survive in waters from different lakes, even though in these short-term experimental settings (one month) no immediate adverse impacts on the native species could be shown. In growth experiments conducted in Australia, a 2–3-fold greater growth of GM microalgae with changed lipid profiles was reported in river water and seawater from the surroundings of the trial site [80]. In addition, Inoue et al. [85] reported similar survival times of 21 days for GM and wild-type diatoms in freshwater or seawater. Experimental evidence for the hypothesis that GM microalgae may have a selective disadvantage due to the metabolic burden of producing the GM trait is mostly lacking [89].

The fitness of microorganisms is highly dependent on the respective environmental conditions [102]. The specific phenotype, as well as the fitness and survivability of GM microalgae in natural habitats, thus needs to be evaluated under experimental conditions reflecting the specific taxon–trait–environment conditions. This may be challenging, since subsequent changes in environmental conditions may facilitate a later expansion of GM microalgae populations. Furthermore, the phenotypic traits of GM microalgae can be significantly different when grown in open ponds compared to laboratory settings [87].

#### 3.4.2. Gene Transfer to Wild-Type Microalgae, Bacteria, or Viruses

The transfer of novel traits via vertical gene transfer is possible if the respective microalgae is capable of sexual reproduction and wild-type algae of the same taxon are present in the environment. In contrast to *C. reinhardtii*, *Nannochloropsis* sp. reproduce exclusively asexually, which minimizes vertical gene transfer [38,103]. Available evidence indicates that certain genes present in members of the genus *Chlamydomonas* originated from horizontal gene transfer (HGT) [104]. As suggested by Snow and Smith [105], Henley et al. [106] and Beacham et al. [35], HGT between microalgae and a range of other organisms such as bacteria or viruses and even animals needs to be considered during ERA.

#### 3.4.3. Adverse Effects on Natural Communities, Food Webs, Biodiversity, and Ecosystem Services

Microalgae form the basis of aquatic and soil ecosystems and food webs and support important ecosystem services [106]. The biotic interactions of many microalgae species, including *C*. *reinhardtii* and *N. gaditana*, with their natural environment are largely unknown [43]. Microalgae are an important food source for microzooplankton such as copepods or planktivorous fish. *Paraphysomonas imperforata*, a flagellate grazer, is able to control *Nannochloropsis* when assessed under experimental conditions [38]. Microorganisms may engage in complex environmental interactions of different types [58,107], such as symbiotic interactions, e.g., the mutually positive relationship between microalgae and nitrogen-fixing bacteria, or antagonistic relationships, e.g., when bacteria inhibit algal growth or exert algicidal effects as indicated in a biology document published by the Australian Office of the Gene Technology Regulator (OGTR) [38].

OGTR [80] found no evidence for increased acute toxicity of *N*. *oceanica* with an altered fatty acid profile. However, a changed nutritional value and the palatability of the GM microalgae and its consequences on predation must be taken into consideration and resulting effects on aquatic food webs and biodiversity. The specific composition of lipids is important for grazers and early larval stages of mussels [35]. The C:N and C:P ratio, as well as long-chain, polyunsaturated fatty acids, are essential for the nutritional value of microalgae for copepods [108]. Changes in the fatty acid composition which affect the relative ratios of carbon, nitrogen, and phosphorous may reduce the food quality for zooplankton grazers in aquatic ecosystems due to the high carbon content of the GM microalgae [80,94,106]. The reported increase in C:N ratio in GM *N. gaditana* is thus likely to shift predation from GM microalgae to other taxa. This can significantly influence trophic dynamics under natural conditions. Under stress conditions or upon cell lysis after algal blooms, fatty acids are released from microalgae into the environment [94,109] and some of these fatty acids have antimicrobial effects [110].

### 3.5. Relevant Risk Issues for the ERA of GM Bacteria Used as Biofertilizers

Based on the growing knowledge on microbial life in soils and the recent advances in microbial biotechnology, the discussion regarding application of GMMs to improve agricultural soils and specifically to fix atmospheric nitrogen to support crop growth has intensified [111,112]. Some GMMs for use as biofertilizers have already been commercialized in some countries, including the USA and Brazil [56,113,114]. Only limited information is available concerning the risk assessment of these GMMs, partly due to the fact that no regulatory assessments were conducted for GMMs developed by genome editing which are not subject to the respective biosafety laws. Another factor is that the information, e.g., regarding the molecular characterization of these GMMs, which was submitted to the authorities for the determination of their regulatory status is considered confidential business information and thus not publicly available. The following discussion regarding the issues for the ERA of GMMs as biofertilizers is based on the data published in the scientific literature [54,56,97], including a commercially used GMM product [56]. This information particularly focuses on (1) the phylogenetic characterization of the bacterial strains, (2) the acute toxicity and pathogenicity of the GMM, as well as the potential for eye and skin irritation in test animals, (3) the stability of the formulated GMM product prior to environmental release, and (4) the nitrogen-fixing ability of the GMO after release into the rhizosphere of plants and effects on the yield of treated maize crops [56].

The relevant risk aspects for GMMs used as biofertilizers are summarized in Table 3.

**Figure 2:**
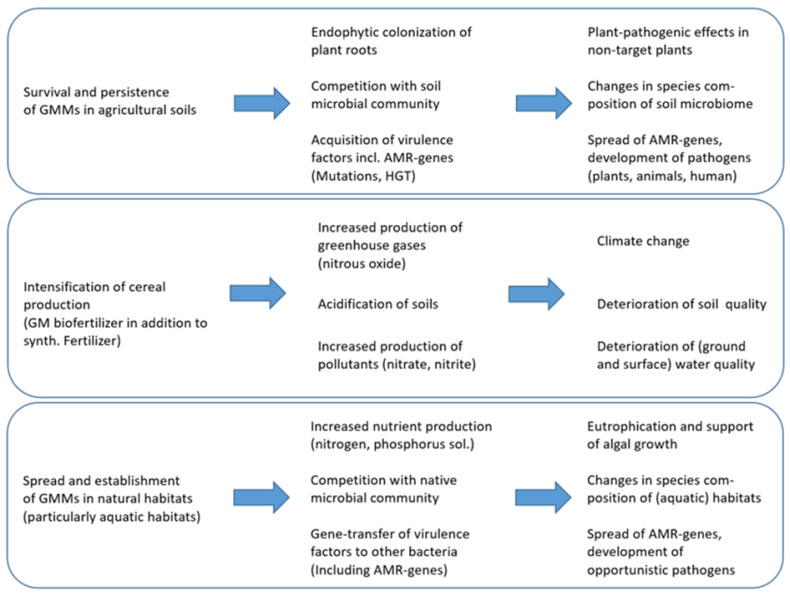
Possible pathways to harm resulting from the intended exposure of agricultural soils and the unintended and unavoidable exposure of natural habitats with GMMs from biofertilizer products. (AMR: antimicrobial resistance; HGT: horizontal gene transfer).

The possible pathways to harm resulting from either the intended release of GMMs as biofertilizers into agricultural soils or the unavoidable secondary exposure of the environment are depicted in Figure 2 and further discussed in the following subsections.

#### 3.5.1. Fitness, Survival, and Persistence of GM Bacteria from Biofertilizers in Natural Habitats

GM *Azotobacter* and GM *Klebsiella* could survive and thrive in the rhizosphere after their release into agricultural soils as well as in other habitats upon dissemination, such as aquatic habitats [54,67]. The modifications introduced by genome editing into diazotrophic bacteria, e.g., by Wen et al. [56] and Bloch et al. [97], result in an increased expression of endogenous nitrogenase enzyme and to an increased rate of ammonia synthesized from atmospheric nitrogen. Biological N-fixation, however, is a very energy-intensive metabolic process that may confer a fitness cost which is associated with the expression of the respective GM trait. GM *K. variicola*, which is associated with crop plants in the rhizosphere, can utilize plant root exudates as an energy source [56]. When crops provide a sufficient amount of carbohydrates to the GM bacteria, no negative selection pressure will result from the GM trait.

Currently, there are only limited empirical data addressing the fitness, stability, and persistence of the respective GMMs in the exposed environments. Short term in planta tests indicate that the used GM *K. variicola* (Kv137-1036) is sustained for up to 10 days by root exudates without an additional carbon source. No longer term evaluation of the respective GM *K. variicola* strain with regard to expression of the intended GM trait was conducted [56]. Initial field tests by Bloch et al. [97] of GM *Kosakonia sacchari* indicated that 12 weeks after their inoculation, genome-edited, ammonium excreting GM *K. sacchari* could be re-isolated from maize roots. Data addressing the establishment and persistence of GM diazotrophic bacteria such as GM *A. vinelandii* and GM *K. variicola* in other, e.g., aquatic, environments are lacking. The ability of *Azotobacter* cells to form cysts that can stay dormant for up to 10 years [115] raises the possibility that such bacteria can endure unfavorable environmental conditions for significant timespans and may expand into larger populations if the resources to sustain rapid growth are available again. Other microorganisms, such as microalgae, can improve the survival und growth of diazotrophic bacteria [58,63], which is also relevant for the ERA of GM biofertilizer applications.

#### 3.5.2. Genetic Stability and Horizontal Gene Transfer

Genetic and phenotypic stability is important for ensuring the establishment of GMMs in the intended receiving environments, i.e., the soils used for agricultural crop production. Genetic stability is particularly an issue for organisms like unicellular bacteria with short generation times and the ability for the rapid expansion of populations under nutrient-rich conditions [115]. The potential for rapid genetic changes is further increased in bacterial strains which display higher mutation rates due to defects in their DNA mismatch repair systems. Such “hypermutator phenotypes” have been described for a number of plant growth-promoting soil bacteria [116], as well as *Klebsiella* strains isolated from clinical settings, such as *K. pneumoniae* and other antibiotic-resistant strains of enterobacteria [117]. Hypermutator traits may also evolve in agricultural settings, which provide new niches for soil bacteria and thus new opportunities to evolve, adapt, and spread. Unfortunately, the specific environmental conditions which facilitate the emergence of hypermutator strains in agricultural environments are only poorly understood yet [116].

HGT between different soil bacteria is also a relevant factor for genetic change and adaptive evolution in soil bacteria like *Azotobacter* and *Klebsiella*. Particularly for diazotrophic bacteria which are able to form dormant forms that sustain longer periods, such as *A. vinelandii*, the uptake of DNA sequences from the environment or the transfer of DNA by HGT is important for evolutionary processes on a longer time-frame [118]. Genetic analysis of *A. vinelandii* indicates that a high proportion of its genes which distinguish *A. vinelandii* from their closest relatives like *Pseudomonas*, including many essential genes, were acquired through HGT and uptake of DNA from the soil seed bank [115,118]. Also, *Klebsiella* sp. is known for the ability to exchange genetic material via HGT [116]. A study by Duran-Bedolla et al. [119] indicated that *K. variicola* can acquire plasmids from a range of enterobacteria species. Larger size, multireplicon plasmids are common in *K. variicola* isolates from medical as well as in environmental samples, e.g., isolated from plants. Such plasmids can harbor resistance factors to various antibiotics as well as virulence genes. These may be passed on to other bacteria including *Escherichia coli*, *Salmonella enterica*, *Pseudomonas aeruginosa*, and *Burkholderia cepacia* [120,121]. Plasmids which increase the virulence of transformed, non-pathogenic strains were also identified in environmental samples of *Klebsiella* sp.; however, they were more frequent in medical isolates [119].

In general, the accumulation and possible spread of antibiotic resistance genes in soils is favored by a disruption of microbial soil communities and a lower level of diversity in such communities [122]. Since the HGT of these factors can promote the transition of commensal soil bacteria to strains which are opportunistic pathogens, a thorough assessment of the potential pathogenicity of plant-growth promoting bacteria prior to their environmental use is recommended, e.g., by Tariq et al. [116].

#### 3.5.3. Adverse Effects on Natural Communities, Biodiversity, and Ecosystem Services

Nitrogen-fixing diazotrophic bacteria, including *Azotobacter vinelandii* and *Klebsiella variicola*, are important for the microbial rhizosphere community, which is characterized by a high level of competition and the presence of many bacteria, which are facultative pathogens, such as *K. pneumoniae* [116]. It is uncertain whether the introduction of significant quantities of GM *A. vinelandii* or GM *K. variicola* impacts the microbial biodiversity of microbial soil communities, where naturally occurring nitrogen-fixing bacteria are typically only present at low abundance [111]. The introduction of significant quantities of GM diazotrophic bacteria could lead to shifts in the soil microbiome if the released GMMs persist for a longer time and can compete successfully with native soil bacteria, as suggested by Wen et al. [56] and Bloch et al. [97].

Benign bacteria may evolve into plant or animal pathogens under conditions of intense microbial competition and close interaction with other opportunistic bacteria [116]. A number of reports indicate the occurrence of pathogenic strains of *K. variicola* in agricultural ecosystems, either as agents causing plant diseases, e.g., in bananas and carrots [69], or as animal pathogens causing, e.g., bovine mastitis [123]. Until recently, the ability of bacteria such as *K. variicola* to cause disease has probably been underestimated due to the difficulties to exactly identify the respective species and strains [69,123].

The nitrogen-fixing ability of GM biofertilizers could contribute to adverse impacts, which are related to an excessive introduction of nitrogen into agricultural soils, particularly if the amounts of synthetic fertilizer used are not reduced appropriately to account for the additional input by the GM biofertilizer. Excessive soil nitrogen may be lost by leaching into water bodies or by bacterial oxidation as greenhouse gases such as nitrous oxide, which is 300 times more potent than CO_2_ [56,111]. Thus, the additional input of nitrogen by GM biofertilizers could lead to an increase in greenhouse gas emissions or to an increased production of environmental pollutants, e.g., nitrate. Such pollutants may also be introduced into non-agricultural environments, in particular into aquatic environments. This can compromise the protection goals directed to the reduction in inorganic pollutants such as nitrate in soil and water, as set by the EU Nitrate Directive 91/676/EEC.

#### 3.5.4. Adverse Effects on Human Health

*Klebsiella* species such as K. *pneumoniae* are known as environmental pathogens, which can cause mastitis in cows and severe infections in humans [123]. Related generalist species such as *Klebsiella variicola*, which can successfully inhabit diverse environmental niches can also colonize human tissues and organs. This may lead to infections and disease particularly in immune-compromised and hospitalized humans [116]. *K. variicola* is regarded as an opportunistic human pathogen and as an agent which causes nosocomial infections [69,116]. At a genetic level, the virulence of *K. variicola* strains is determined by genetic adaptation to the human host and the acquisition of plasmids, which express virulence factors like toxins, adhesins, or resistance factors against a range of commonly used antimicrobial drugs [116]. Similar as for *K. pneumoniae*, hypervirulent strains of *K. variicola* were detected, which can cause severe infections with an increased mortality in apparently healthy humans [124]. The parental strain of *K. variicola*, which was used to develop *K. variicola* 137-1036 belongs to a phylogenetic clade that is distinct from *K. pneumoniae*; however, no systematic analysis of virulence factors was conducted by Wen et al. [49].

### 3.6. Adequacy of the Existing EU Guidance for the ERA of GMMs

Currently, the ERA of GMMs in the EU is based on Directive 2001/18/EC and several EFSA guidance documents. These include the EFSA guidance on the risk assessment of GMMs and their products for food and feed use [76] and a number of opinions and guidance documents issued by the GMO panel or other EFSA panels (for an overview, see [78]). Recently, the EC tasked the EFSA to evaluate the existing EU guidelines for their adequacy regarding the microbial characterization and ERA of GMMs obtained through synthetic biology [77]. Subsequently, the EC requested the EFSA to assess whether the available EFSA guidelines are applicable and/or sufficient to address the risks of GMMs developed by new genomic techniques, such as genome editing [78]. EFSA, however, limited the scope of their assessments to GMMs used in food and feed or for the production of crop plants. Thus, for GM microalgae the release of viable GMMs into the environment was not considered by the EFSA.

In general, we agree with the EFSA [77,78] that the existing risk assessment guidance in the EU is not sufficient for applications which include the environmental release of viable GMMs (category 4 applications according to EFSA [125]). We also support the recommendation by EFSA that new and/or updated guidance should take a consistent approach for all modified microorganisms, irrespective of the technique used for modification [78]. We agree that the risk assessment approach should focus primarily on the characteristics of the particular GMM and its interactions with the environment. Issues which need to be addressed by updated guidance are outlined in the following sections.

#### 3.6.1. A Comparative Assessment May Not Be Applicable for Certain Types of GMMs

A comparative assessment may be challenging for certain types of GMMs due to the lack of knowledge on the biology and ecology and therefore the safety of the parental (non-GM) taxon. This is also true for synthetic genes, gene fragments, or regulatory elements generated by artificial intelligence. A history of safe use is lacking for a range of GMMs, such as GM microalgae (*N. gaditania*) and GM bacteria (*K. variicola*). The existing guidance does not require information on phenotypic traits that may affect the survival and reproduction of a GMM in different environments. The biological and ecological characterization of the respective microorganism should therefore be specified in the guidance to provide a the basis for a comparative assessment. This includes information on, e.g., habitat requirements and growth characteristics under relevant environmental conditions, the host range of symbiotic or pathogenic microorganisms, and other aspects such as photosynthetic ability, production of pigments, N-fixation ability, temperature range, and salinity tolerance for microalgae [94,126]. If such information is not available for a specific taxon, the applicability of a comparative approach must be questioned. In such a case, an assessment approach which does not require an adequately characterized comparator must be developed.

#### 3.6.2. The Guidance for Microbial Characterization Needs to Be Updated

EFSA recommends that whole genome sequencing (WGS) should be used for the taxonomic identification and characterization of the respective microorganism species as well as for the assessment of the genetic modification(s) in a GMM [77,78]. However, the technical limitations for WGS and the difficulties in interpreting WGS data need to be taken into account when assessing such data. The current approaches are not sufficient to comprehensibly identify virulence factors and other genes of concern or to reliably determine the taxonomic identification of certain taxa, including microalgae and soil bacteria. For taxonomic identification, multipronged approaches are recommended for soil bacteria [116] and eukaryotic algae [126] including genomic data (16S rRNA sequencing, WGS and multilocus sequence typing), phenotypic assessments, chemosystematics, and morphological characteristics, as available.

#### 3.6.3. The Guidance for the Molecular Characterization of the GMM Needs to Be Updated

According to the EFSA, WGS approaches should also be used for the assessment of the genetic modification(s) in a GMM and its genetic stability [77,78]. However, the existing information requirements in the current EFSA guidance [76] refer only to the genetic stability of GM traits during the production process under contained use conditions.

Currently, no specific guidance is available to assess the genetic and phenotypic stability of GMMs upon environmental release and long-term persistence. The stability of the phenotypic trait is ecologically highly relevant and genetic instability of traits is often observed in GMMs [127]. Due to their short reproduction cycles, the evolutionary changes after release are by far more relevant for GMMs than, e.g., for GM higher plants.

#### 3.6.4. The Guidance for the Assessment of Health Effects (Toxicological, Allergenic, and Pathogenic Effects) Needs to Be Further Developed

Indirect exposure of animals and humans to GMMs through soil, contaminated plant material, or water may possibly lead to allergenic effects or adverse impacts on the gut microbiome. The EFSA GMO panel recommended that guidance and assessment methods should be developed to address the human gut microbiome as well as for adjuvanticity regarding potential allergenic effects [78]. 

As concluded by More et al. [77], suitable testing systems for virulence and pathogenicity of microorganisms are not yet available and need to be developed. This is particularly challenging for GMMs such as GM biofertilizers which are released repeatedly on a large scale over a longer time and likely disseminated into different environments [57].

#### 3.6.5. Guidance for the Assessment of Ecotoxicological Effects Needs to Be Developed

In the case of GM microalgae with a changed fatty acid profile, the assessment of potential toxicological effects of the altered composition on organisms is important. However, tests for acute toxicity as recommended by US-EPA [126] cannot predict the effects resulting from a chronic, long-term exposure. In addition, these tests generally use standard test organisms (e.g., *Daphnia magna*) and are not targeted to assess impacts on other microorganisms as well as indirect effects on higher trophic levels in the affected habitats. Testing approaches for environmental effects of GMMs should consider a well-developed set of relevant non-target organisms, which are representative for the habitat types likely to be exposed. For this purpose, selection procedures, test systems, and protocols have to be developed that address ecotoxicological as well as nutritional effects on non-target organisms due to the changed composition of the GMMs [94].

#### 3.6.6. The Exposure Assessment Needs to Address All Potential Receiving Environments

The current guidance needs to be revised to address all possible pathways of environmental exposure due to both intended releases, e.g., of GM biofertilizer agents, or unintended releases, e.g., of GM microalgae, which are either unavoidable due to the design of the production facilities or may happen accidentally. EFSA also recommends addressing primary and secondary routes of environmental exposure by GMMs; however, only the exposure of the environment to manure from farm animals that consume feedstuffs produced from GMMs is highlighted as a route for secondary exposure [66]. Other relevant possible scenarios of secondary exposure are, e.g., the exposure of farm animals and humans to GM biofertilizer agents and the accidental exposure of soil and water bodies to GM microalgae from open pond production facilities. The assessment of secondary exposure pathways is further complicated by the fact that the level of exposure to viable GMMs, which can reproduce with varying velocities, cannot be predicted with sufficient certainty without data from monitoring such releases.

#### 3.6.7. The Guidance for ERA Needs to Be Updated

EFSA indicated that the currently existing suite of guidance documents for GMMs is not sufficient to inform the ERA addressing the release of viable GMMs into the environment (i.e., category 4 GMMs). EFSA concludes that the existing guidance neither covers all receiving environments nor addresses all ‘specific areas of risk’ as required by Directive 2001/18/EC [77,78]. An updated ERA guidance in our opinion also needs to consider the biological characteristics of microalgae which are different from those of other microorganisms. This concerns aspects such as their photo-autotrophy, their ability for gene transfer by sexual reproduction, their specific growth requirements, and ability to occupy specific, but not well characterized, ecological niches [12,101].

The limited knowledge of the ecology of most microorganisms, including microalgae, impedes the ERA of GMMs. Hence, methods, such as microcosm or mesocosm experiments, need to be established to assess relevant aspects, such as the competitive advantage, the potential survival and persistence of GMMs and the transfer of genes and traits from GMMs to other microorganisms [105,128,129]. The assessment of effects on ecosystem functions and services should also be integrated into the ERA [77,82]. Further guidance also needs to be developed for the transfer of genes of concern and GM traits to other (micro)organisms and the potential acquisition of virulence and pathogenicity factors by GMMs via HGT.

#### 3.6.8. Specific Guidance for PMEM of GMMs Needs to Be Developed

We agree with EFSA that fit-for-purpose approaches to monitor potential adverse effects resulting from environmental releases of GMMs and specific guidance for PMEM of GMM applications should be developed. This includes adequate guidance for the general surveillance of GMMs [77,78], and guidance to assess and monitor long-term environmental effects. Specific monitoring approaches and methodologies need to be developed and existing monitoring networks e.g., on an EU member state level, should be scrutinized whether these could be harnessed for the PMEM of GMMs. Existing national environmental monitoring schemes, e.g., according to the Water Framework Directive (Directive 2000/62/EC), cover a range of chemical and ecological parameters, including aquatic species, which are relevant for the PMEM of GM microalgae [23]. The assessment of an ecological baseline status of microbial communities, as a reference point for the monitoring of potential adverse environmental effects of GMMs, is particularly challenging due to the dynamics of species composition and abundance [90,101]. The use of tools such as the German Environmental Specimen Bank [130] could help to address such challenges. However, practical challenges associated with the monitoring of environmental releases of GMMs, e.g., regarding the availability of appropriate methods and the costs of implementing such measures, need to be further explored. Approaches for technical standardization of monitoring as developed for GM plants [131] could be beneficial for overcoming the significant technical and resource challenges related to the PMEM of GMMs.

## 4. Considerations for a Broader Assessment of GMM Applications Beyond ERA

The existing regulatory framework in the EU for GMOs in general and GMMs in particular does not contain concrete requirements for a broader assessment, e.g., by a sustainability analysis or through technology assessment (TA). In addition to the mandatory risk assessment, broader considerations may be taken into account according to Directive 2001/18/EC and Directive (EU) 2015/412, the latter allowing for measures by EU member states to restrict the cultivation of GM crops in their territories. However, no concrete guidance is yet available how such a broader assessment should be carried out [132], neither for a sustainability analysis nor for TA of GMM applications.

### 4.1. Sustainability Analysis of Environmental GMM Applications

The use of GMMs, including microorganisms that were genetically modified by genome editing methods, is frequently aimed at improving the efficacy and sustainability of modern agricultural production and other areas, such as the production of renewable raw materials and fuels [133]. The proposed goals are wide-ranging and often rather general, including among others the further improvement of yield and the quality of major crops and the sustainable production of foods and fuels, while reducing negative impacts, e.g., on climate, land use change, and (environmental) health. Both of the case studies discussed in this paper are associated with some of these objectives.

According to the objectives of the European Green Deal developed by the European Commission and the Farm-to-Fork Strategy, the EU is aiming towards a transition to a more environmentally friendly agriculture [134]. This has spawned discussions how to consider aspects beyond health and environmental risks under the EU legislation on GMOs [135]. In contrast, other fields of EU regulation, such as chemicals regulation according to REACH, have established both an institutional framework and guidance to address socio-economic considerations and thus issues related to sustainability [136].

At the global level, the Cartagena Protocol on Biosafety, which regulates GMOs on the UN level [137], allows the parties to the CPB to voluntarily take into account socio-economic considerations in their decisions. These considerations may address issues from all three dimensions of sustainability (social, environmental, and economic sustainability) [138]. Voluntary guidance on the assessment of socio-economic considerations in the context of the CPB was developed in 2018 [139]. Additionally, proposals for a socio-economic analysis of GMOs were developed by various European expert bodies. These include, e.g., considerations for an EU sustainability assessment of GM crops by the Dutch Commission on Genetic Modification [140], criteria for a socio-economic analysis of GMOs by the French High Council for Biotechnologies [141], and a framework for socio-economic analysis of GM crop cultivation by the European Socio-Economics Bureau [142,143].

In Norway, sustainability issues are explicitly covered by the Norwegian GMO legislation, which includes ethical, social, and sustainability objectives [144]. This has led the Norwegian authorities to reject ten applications for GM plants, which were authorized for import into the EU [145]. Specific guidance for the sustainability assessment is, however, available only for herbicide-tolerant and insect-resistant GM plants [146,147].

Due to the current lack of practical experience with sustainability analysis of GMO or GMM applications in the EU, the following subsections present initial considerations to address basic requirements for an approach for sustainability analysis for GMMs.

#### 4.1.1. Framing of the Sustainability Analysis

##### Case Study 1: GM Microalgae for Biofuel Production (CS1)

Ajjawi et al. [41] suggested that microalgae, such as *Nannochloropsis* sp., may be an important source for the production of biofuels [41], which are not associated with the same negative impacts on food and feed production and security as, e.g., plant-based biofuels [148]. As a viable alternative to fossil fuels such biofuels have to be produced in large quantities, i.e., at a commodity scale level, with a high productivity [149]. The use of GM microalgae with an improved capacity for the synthesis of lipids that can be converted into biofuels is meant to increase productivity and to enhance their competitiveness with fossil fuels or other sources of energy.

##### Case Study 2: GM Bacteria for Use as Biofertilizer (CS2)

The provision of alternatives to synthetic nitrogen fertilizers for cereal crops and an overall increase in the use efficiency of nitrogen fertilizers are important for the transition of modern agriculture to a more sustainable way of production. Wen et al. [56] and Das [54] have suggested that GM *K. variicola* or GM *A. vinelandii* can be used as biofertilizers, which are environmentally safe and economically and socially accessible for all cereal farmers [54,56,111]. They have suggested that GM biofertilizers can partially substitute for synthetic or organic nitrogen fertilizer in cereal production and thus may reduce the damage caused by the excessive use of synthetic nitrogen fertilizers. However, non-GM biofertilizers cannot be used together with other nitrogen fertilizers or in soils with a higher level of residual nitrogen, which are common in cereal production [56].

A framework to assess the sustainability of both case study applications needs to consider all three dimensions of sustainability (environmental, economic, and social sustainability). Within this framework, the specific issues have to be identified and addressed which are relevant for the use of biofertilizers for either the agricultural production of cereal crops (CS 2) or the production of renewable biofuels and by-products that are used mainly in animal feed from GM microalgae (CS 1).

For crop production with or without GM biofertilizers, the SAFA guidelines developed by FAO [150] to address the sustainability of agriculture and food supply chains can be considered as a starting point. A recent analysis of several tools developed for the sustainability assessment of agricultural (dairy) production conducted by Paçarada et al. [151] found that the SAFA guidelines represent an easily accessible tool with a comprehensive coverage of all sustainability dimensions and cover inputs beyond the farm level. The SAFA guidelines outline a general set of indicators to evaluate the sustainable development of agricultural production, addressing the risks and opportunities as well as trade-offs. These indicators can be adapted to better address specific agricultural production systems. For biofuels, the current approaches for Life Cycle Analyses (LCAs) can provide relevant input [152,153]. To further address feed by-products, indicators derived from the SAFA approach can be integrated into the analysis framework. The SAFA approach is also applicable for assessing biofuels produced from cultivated plants.

The assessment approach should compare different production systems with or without the use of the respective GMMs rather than comparing a specific GMM product with its non-GM counterpart. This is addressed by selecting appropriate production and reference production systems and by defining the system boundaries for the analysis.

The system boundaries for the sustainability analysis of biofuel production with GM microalgae (CS1) can be derived from previously conducted LCAs, which consider the inputs necessary for production, including water, nutrients and energy, costs for building and operating the production facilities, as well as land use for their development and the energy that is necessary for processing of the cultivated microalgae into biofuel [152,153].

The system boundaries for the sustainability analysis of GM biofertilizers (CS 2) should include the inputs to and outcomes of relevant cereal crop cultivation systems, with a particular focus on the inputs and the effects of the respective nitrogen fertilization regimes. This includes the effects, including socio-economic effects, of the organic and synthetic nitrogen fertilizers as well as the biofertilizers used in the specific scenarios.

Another critical step for the sustainability analysis is the definition of the scenarios that are compared, i.e., the test and reference production systems addressed by the analysis. These scenarios should correspond to the proposed use of the GMMs in question and current and foreseeable alternative approaches which are not based on GMMs. Table 4 provides an overview on the test and reference production systems for both case studies.

For the production of biofuels from GM microalgae, different systems are used, which can be assessed as test production systems (TPS) based on their respective characteristics:

One the one hand, open pond or semi-open pond systems (e.g., raceway ponds) can be used for (commercial) production of microalgae (TPS1). Production in open ponds enables the production of high volumes; however, this typically requires large areas of land (up to 10.000 ha or more) and does not provide a full containment against the accidental introduction of GM microalgae into the environment. These facilities are technically simple and comparably cheap to build and operate [35,152], as less human intervention is needed to supervise and maintain production. However, not all microalgae are suited for this type of production, some yield potential can be lost, and the production is prone to contamination, depending on the respective environmental conditions [152].

Alternatively, microalgae may be cultivated in closed-culture systems, such as bubble column photobioreactors and tubular photobioreactors [154]. Such facilities which are suggested as TPS2 typically are more costly to build and operate than open ponds, but provide higher yields due to better control of the production environment [152]. The design may integrate different production stages, such as heterotrophic production in lower-cost fermenter systems coupled with auxotrophic growth of microalgae in photobioreactors to increase efficiency [153].

As reference production systems (RPSs), the production of biofuels from non-modified microalgae and the production (RPS1) and use of fossil fuels (RPS2) could be used for comparison. The analysis may be broadened by further considering plant based-biofuels or other renewable energy sources (e.g., solar, wind) as additional comparators.

For CS2, two possible use scenarios for GM biofertilizers are suggested by the developer [155] and discussed in the literature [56]:

On the one hand, GM biofertilizers such as GM *K. variicola* KV137-1036 (ProveN 40™) may be used together with the fertilization regime with synthetic nitrogen fertilizers or organic fertilizers as applied in intensive maize cultivation (TPS1). This results in a slight yield gain of the maize crop (3%) and a significant reduction in yield variance at treated plots in comparison to untreated ones [56]. It is uncertain whether this results from the additional nitrogen introduced by KV137-1026 or other growth promoting effects of the GMM.

On the other hand GMMs as biofertilizer may replace a fraction of the treatment of maize crops with synthetic nitrogen fertilizer (TPS2). According to Pivot Bio, a reduction in nitrogen fertilizer of 7.2 kg N (urea)/ha is possible for maize cultivation upon treatment with ProveN 40™, and smaller reduction rates for wheat and sorghum (4.5 kg N (urea)/ha) [155]. At best, about 20% of the total synthetic nitrogen fertilizer used in the USA as standard growing practice may be substituted by the treatment with the GMM [133].

For comparison, two other cereal production systems may be used, including a standard scenario according to the current agricultural practice for cereal production with high inputs of synthetic and/or organic fertilizers (fertilization levels of up to 220 kg N (urea)/ha in the USA) (RPS2) and a less intensive production scenario that is based on biofertilizer use only, without any added organic or synthetic fertilizers (RPS1). In the latter scenario, the use of non-GM biofertilizer is feasible. However, biofertilizers developed from non-GM soil bacteria do not provide sufficient plant-available nitrogen to sustain the current yields of the major cereal crops, e.g., maize and wheat [111] GM biofertilizers could lead to a slightly increased crop performance, if the respective GM product is offering higher rates of nitrogen fixation in soils with a low nitrogen content.

#### 4.1.2. Issues to Be Addressed by the Sustainability Analysis

The SAFA guidelines [150] provide a general framework to address sustainability issues relevant for GMMs. The framework can be adapted for the analysis of different GMMs, such as GM microalgae and GM biofertilizer agents, by the selection of appropriate themes, subthemes, and indicators. Table 5 provides an overview of the dimensions and the themes for a sustainability analysis of GMMs, indicating relevant subthemes for the case studies. A brief discussion is presented in the following subsections.

##### Sustainability Issues Relevant for GM Microalgae for Biofuel Production

The most important aspect of the ecological dimension is whether the production of biofuels can achieve a reduction in the emissions of greenhouse gases compared to the use of fossil fuels, which may not be the case for less efficient production systems [153]. Direct energy (electricity) necessary for the production process and land use for infrastructure are major sources of environmental impacts, together with the amount of water that is necessary for large-scale biofuel production. Large-scale non-fully contained production may also impact the water quality and biodiversity of (semi-)natural water bodies which are exposed in case of accidental spillage of GM microalgae (cf. Section 3.4). If GM microalgae are cultivated using wastewater as medium, a net positive outcome, including an improvement in the quality of the treated waste may be achieved. However, such facilities would not focus on the production of biofuels as their main goal, but consider them as by-product of the wastewater treatment [157]. Land use for building the necessary infrastructure needs to be considered as a major environmental impact. Significant areas of land are required, particularly for the large-scale production of biofuels from GM microalgae.

Substantial investments into research and significant costs are required for the development of an up-scaled, efficient production system for biofuels from GM microalgae [158], in addition to the investments into infrastructure to improve productivity [153].

In the social dimension, the production of sustainable biofuels can support policies to advance the energy transition to more sustainable fuels and help to address the challenges of climate change [153], while supporting the necessary levels of human mobility and create a multitude of job opportunities in research and production.

##### Sustainability Issues for GM Biofertilizers

Regarding the environmental dimension, the effects of a continuous introduction of small amounts of bioavailable nitrogen in the vicinity of plant roots by GM biofertilizers could be beneficial to decrease nitrogen loss from the soil due to runoff, leaching, acidification, or denitrification and volatilization [159]. Also, a significant reduction in use of synthetic fertilizers would be beneficial regarding the degradation of agricultural soils, soil health, the microbial biodiversity, as well as for water quality and could reduce the emission of greenhouse gases. However, the potential reduction in TPS2 is rather small in comparison to the current levels of nitrogen fertilizer use in cereal crops. TPS1, on the other hand, offers only economic incentives, but no beneficial environmental effects as the amount of nitrogen introduced is even higher than in the standard scenario based on the current use of nitrogen fertilizers. As a sole measure, the use of GM biofertilizers would fall short of achieving the EU policy targets for nitrogen fertilizer reduction.

A complete substitution of all synthetic fertilizers with GM or non-GM biofertilizers would be more beneficial in terms of the environmental effects but would likely lead to yield and income reductions compared with the current revenue generated by intensive cereal crop cultivation. Use of GM biofertilizers could also partly decrease the current dependence on synthetic fertilizers and thus benefit users in industrial agricultural systems, particularly if the costs of synthetic nitrogen fertilizers increase in parallel with rising energy prizes. Also, supply problems regarding synthetic fertilizers could be alleviated to some extent, particularly in parts of the world where synthetic fertilizers are less accessible and the cost of the used fertilizers is a decisive factor [57,160]. Biofertilizers may be able to increase productivity in marginal agriculture without a use of synthetic fertilizers.

Better income security and less dependence on external inputs such as synthetic nitrogen fertilizers can improve the livelihood of small-holders and marginal farmers in the social dimension; however, it needs to be ensured that no health and safety effects for farmers, farm workers, and farm animals are associated with (GM) biofertilizers. The use of GM biofertilizers also poses challenges for governance, e.g., to develop management practices for GM biofertilizer products to enhance the overall efficacy of fertilizer use.

### 4.2. Broader Consideration of Governance Issues Raised by GMM Applications by Technology Assessment (TA)

Historically, technology assessment (TA) was originally developed to advise a parliamentary body. The Office of Technology Assessment (OTA) was established in 1974 at the U.S. Congress as the first TA institute and has served as an influential model for later implementations of TA, mostly in Europe [161]. Many contemporary TA institutions report directly to national and transnational parliaments, like the Parliamentary Office of Science and Technology (POST) in the United Kingdom, the Office of Technology Assessment (TAB) at the German Bundestag, the Parliamentary Office for Scientific and Technological Assessment (OPECTS) in France, or the Panel for the Future of Science and Technology (STOA) at the European Parliament. The Austrian Institute of Technology Assessment (ITA), formally part of the Austrian Academy of Sciences, also answers to the Austrian parliament based on a framework established in 2017.

The paradigmatic task of TA is to advise parliaments, which are public bodies engaged in socio-political debate and political institutions invested with legislative power. TA thus essentially serves the purpose of ‘opening up’ emerging issues regarding new technologies and their applications for public scrutiny, rather than ‘closing down’ public debate based on already established regulatory frameworks [162]. In this way, TA differs fundamentally from ERA regarding the type of scientific policy advice which is provided.

Against this institutional background, it is not surprising that many TA reports bring up a much broader array of issues than risk assessment or even sustainability assessment, encompassing ethical, legal, social, cultural, ecological, health, and economic dimensions. In most cases, TA reports feature a comprehensive aspiration, aiming at a full set of (potential) issues raised by a specific socio-technological innovation; only in a few cases, TA reports focus on a single dimension or issue—in response to the respective parliament’s interests and assignment. In general, TA practitioners aim at covering all potential intended and unintended ramifications that are considered to be societally relevant [163] (compare the International Risk Governance Council’s (IRGC) broad definition of risk as “refer[ing] to uncertainty about and the severity of the consequences of an activity or event with respect to *something that humans value*” (emphasis by the authors)). Moreover, they will do so as early as possible so as to guide technology governance from the early stages of innovation, limiting harmful effects as well as regulatory costs and preventing technological lock-ins [164]. Finally, most TA institutes do not cover all socio-technological innovations at all times. With limited resources and an abundance of socio-technological innovations, they have to be selective. These general characteristics of TA may explain the ways in which TA addresses the environmental applications of GMMs.

**Figure 3:**
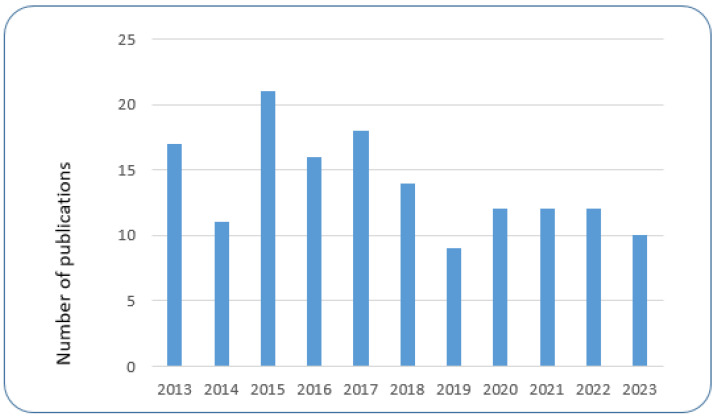
Number of topical publications per year (n = 155).

Based on an analysis of the publications of eleven European TA institutions (OPECST/FR, POST/UK, ITAS/DE, TAB/DE, Rathenau/NL, DBT/DK, ITA/AT, NBT/NO, SPIRAL/BE, STOA/EU, TA-SWISS/CH) between 2013 and 2023, we find that the overall coverage of biotechnology, including genetic engineering, has slightly decreased (see Figure 3), despite the continued technoscientific progress in this field.

**Figure 4:**
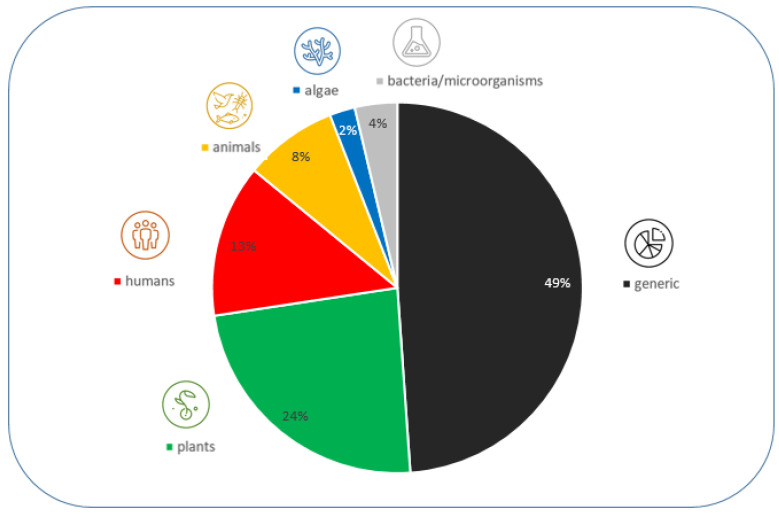
Topical split within the analyzed sample of publications (n = 135).

One explanation might be that resources available to TA institutions did not increase, while the demand for TA of different socio-technological innovations, e.g., in the context of digitalization, but also energy technologies, has rapidly increased in the past decade. Another factor is presumably that TAs tend to specifically focus on major technological breakthroughs early after these inventions, such as new genome editing techniques, have been made. As a consequence, TA reports tend to be rather generic, discussing a technological innovation in general rather than specific applications in distinct contexts. This pattern is visible in Figure 4 depicting the organisms which are at the focus in the analyzed TA reports. Half of the publications share a generic focus on biotechnology (and particularly on genetic engineering), while only the others address particular (GM) organisms and, thus, the distinct contexts of their application. Only 4% of the studies focus on bacteria and other microorganisms; some publications with a generic focus mention this group of organisms inter alia.

For a qualitative analysis of TAs addressing GMMs, we are thus left with three publications primarily focusing on microalgae [165,166,167] and nine publications with mentions of GMMs inter alia [168,169,170,171,172,173,174,175]. The issues addressed in these publications span from biodiversity, ecosystem conservation, and sustainability to public perceptions, public discourse, stakeholder engagement, and public participation in opinion formation and decision making. They address public welfare, economic viability, responsibility, accountability, transparency, ethics, and unknowns associated with GMM applications.

Regarding the two case studies addressed in this paper, by far more expertise is provided on GM microalgae for biofuel production than on nitrogen-fixing GM soil bacteria for use as biofertilizers. Three TA institutes, ITAS and TAB in Germany as well as the Rathenau Institute in the Netherlands, have addressed the use of oil produced from GM microalgae [165,166,167]. Asveld and Stemerding [165], however, focused on oil from GM algae as a basic ingredient in consumer cleaning products.

Schröter-Schlaack et al. [166] authored a comprehensive report for the German Parliament on the potential of algae-based fuels for trucks. GM algae are referred to in various sections of their report. They emphasize that microalgae populations exhibit a high biochemical variability, reacting strongly to environmental conditions, and argue that targeting distinct characteristics by genetic modification seems not very promising given the short reproduction cycles and high adaptability of the used algae and the potential to use non-GM strains with a higher productivity (see Schröter-Schlaack et al. [166], cf. Refs. 43 and 129 therein). This would render the use of GM algae economically unattractive. They add that robust containment was rather unrealistic when up-scaling production systems, posing risks related to the unintended release of GM microalgae (see Schröter-Schlaack et al. [166], cf. Ref. 129 therein). For algae-based fuels in general, the authors refer to claims regarding reduced land consumption as compared to other biofuels, but state that potential advantages discussed in the literature have not yet been realized in practice. Also, robust evidence for large scale production systems was missing, despite the dedicated research efforts over the past 80 years. They also mention the water and energy consumption of algae production systems, leading to water dependence and overall negative energy yields. They conclude that the use of algae-based biofuels will probably not lead to a relevant reduction in traffic-induced greenhouse gas emissions until 2050.

Varela Villarreal et al. [167] reported on the acceptability of biofuels from GM algae in Europe for experts and stakeholders. Their survey addressed the relative advantages of GM algae biofuels as compared to fossil fuels and biofuels from non-GM algae and other natural resources. They found high expectations for several advantages, including reduced import dependency, the creation of new jobs in rural areas, reduced greenhouse gas emissions and less environmental impact in comparison to fossil fuels, lower competition with food production in comparison to established biofuels, and improved productivity in comparison to biofuels from non-GM algae. The surveyed experts, however, did not expect differences in engine performance in comparison to established biofuels. GM algae biofuels were in general deemed to be much less risky than fossil fuels and even established biofuels, but still riskier than alternative energy sources such as photovoltaic, wind or hydropower. On the other hand, public acceptance was estimated as medium to low, and recommendations were given on how to raise public acceptance, highlighting clear evidence-based communication of risks and benefits, closed production systems with high security standards and rigorous, independent and participatory risk assessment. The authors add that “[a]lthough our results indicate a higher preference for GE [genetically engineered] algae biofuel compared to first generation biofuels, it cannot be concluded that people will purchase the product once algae biofuel is on the market, and even pay more money for it, compared to other fuels. Since there is an intensive debate on sustainable mobility in general and a trend to ban cars with combustion engines, it is not surprising that mobility provided by green electricity based on hydro, wind and solar power is regarded as even more desirable due to lack of emissions and climate-friendliness.” But they agree “that fast-track algae biofuel production could be a feasible midterm solution to replace fossil transportation fuels in trucks and airplanes” and highlight that fuels containing algal biofuels positively impact combustion and emission (see Varela Villarreal et al. [167], cf. Ref. 11 therein).

Finally, many TA publications addressing genetic engineering under the label of synthetic biology or genome editing, focus on public attitudes. Exemplarily, Rerimassie and Stemerding [176] reconstruct various framings that motivate critical stances in various publics. They refer to key cultural narratives in public debates on science and technology (e.g., “be careful what you wish for”, “opening Pandora’s box”, “messing with nature”, “kept in the dark” or “the rich get richer and the poor get poorer”), that are thought to be deeply rooted in our culture (based on Macnaghten et al. [177]). The cultural dimension represented by narratives and framings serves an important reference for TA processes that target more broadly what people value, hope, and fear. Asveld and Stemerding [165] highlighted the central role of narrative frames and respective worldviews in the public debate on oil from GM algae as a basic ingredient in consumer cleaning products. However, environmental applications of GM algae and other GMMs have not yet been addressed by specific TA studies in that respect.

## 5. Challenges for the Assessment of GMM Applications, Particularly for ERA, Sustainability Analysis, and Governance of Such Applications

### 5.1. Open Issues for the ERA of GMMs

GMMs are fundamentally different to GM higher organisms. These differences are highly relevant for the ERA and monitoring of GMMs in agricultural and natural ecosystems, as discussed in detail for the two case studies in Section 3.

The microscopic size of bacteria and other microorganisms complicates the detection of their presence in the environment and thus poses methodological challenges for assessing and monitoring of their survival after intentional or unintentional release. Similar challenges also concern the assessment and monitoring of their spread and persistence in different environmental compartments. Furthermore, the existing difficulties concerning their (taxonomic) identification also present challenges to predicting or identifying whether they are associated with a specific (adverse) ecological effect.

Short generation times facilitate the rapid expansion of GMM populations under favorable environmental conditions after inoculation or release and in recovery from low abundance and resting states. GMMs are generally characterized by a high mobility as well as spread and dispersal in soil or aquatic environments. In addition, GMMs may be transported by water, air, or other organisms to ecosystems other than the intended environments, including (semi-)natural habitats. It is also questionable whether GMMs can be removed from exposed environments after their intentional or unintentional release.

Bacteria and microalgae are able to exchange DNA sequences via HGT, including sequences which may confer adverse characteristics (virulence and pathogenicity) or a fitness advantage in comparison to non-modified microbes. Released GMMs may evolve rapidly after release into the environment; their genetic stability and the stability of the phenotypic traits is considered to be lower as for other higher organisms. Genetic stability of the respective GMMs can only be ensured up to the point of release and there is a lack of standardized methods for the assessment of their genetic stability, fluidity, fitness, and persistence; issues which are crucial for assessing the long-term persistence and potential adverse consequences related to the long-term presence of GMMs in the environment.

GMMs, including the case study organisms discussed in Section 3, may impact a range of different ecosystem functions and species in the exposed ecosystems, including animals and their microbiomes [25]. EFSA has highlighted the importance of assessing the gut microbiome for changes induced by GMM food and feed products [78]. For the environmental applications of GMMs, possible effects on the microbial communities of the exposed environments, including the gut microbiomes of wild animals, are an equally important issue. However, it is difficult to assess or predict the effects of GMMs on the microbiome or on the biodiversity of other trophic levels due to the limited knowledge of the functional diversity of microbial communities of ecosystems.

The complex ecological interactions in the receiving environments and the fluctuations in the respective microbial communities are poorly understood, which makes predictions of the adverse ecological effects of GMM highly speculative, particularly regarding their long-term effects. This poses significant challenges for problem formulation and the ability to derive testable risk hypotheses for ERA and the development of appropriate testing and monitoring methods. In particular, this concerns effects, which may arise with significant delays after the initial release of GMMs and due to the potential long-term survival in the environment. In view of these challenges and the limited available knowledge, a high level of uncertainty is associated with predicting the outcomes of GMM releases.

Due to the different characteristics of individual GMM applications and the limited (ecological) knowledge on some GMMs, a case-by-case approach to risk assessment is necessary. This is also stressed by EFSA in their new draft guidance document [79]. Similarly as for GM virus applications and other GMO applications which are associated with assessment uncertainties, a precautionary approach towards the environmental release of GMMs should be implemented [18,178,179].

Measures proposed for physical containment and biocontainment may prevent the unintentional release, the spread and persistence of GMMs in the environment, and gene flow by HGT [25,89]. However, it is uncertain whether such mechanisms (described in Section 3.3.1) are fit for purpose, particularly in the long term and if different types of GMMs are released at the same time.

For devising appropriate guidance for the comprehensive assessment of GMMs, additional examples of different GMM applications need to be considered, as data for their evaluation under real-world conditions become available.

### 5.2. Open Issues Regarding a Broader Assessment of GMM Applications

As indicated in Section 4, there is a need to establish a framework for an evaluation of applications of GMMs for environmental release that goes beyond the current remit of ERA to address the robustness of the presumed sustainability advantages of GMM applications put forward by the developers and to resolve issues concerning the (public) acceptability of such applications. While the former issue could be addressed by sustainability analysis (Section 4.1), TA efforts directed towards GMM applications may help tackle the latter aspect (Section 4.2). However, a number of open issues need to be further addressed to successfully implement such instruments.

To advance the systematic analysis of the sustainability of environmental applications of GMMs, some general issues need to be addressed that are also relevant for a sustainability analysis of all other GMO applications. These include the following:

- The scope and the objectives of a sustainability analysis needs to be defined as a starting point;
- A process to structure such a sustainability analysis has to be devised. This may be best achieved by a multidisciplinary and iterative process as proposed for the sustainability assessment of GM crops by Wohlfender-Bühler et al. [180];
- An institutional framework for conducting sustainability analyses for GMOs, as well as for GMM applications is lacking in contrast to other fields such as EU chemicals regulation (REACH), where a framework for socio-economic assessment was established in 2006 [132,136];
- The nexus to the existing requirements for ERA is not well defined, particularly for issues regarding ecological aspects of a sustainability analysis;
- It also needs to be worked out how the results of a sustainability analysis can be communicated to the regulators and the public.

For the latter issue, an approach is needed to determine how the results of a sustainability analysis can be taken into account during overall decision making and how these results and the uncertainties associated with such analyses can be communicated in a transparent way.

Additionally, a number of open issues which are specific to certain GMMs and to environmental applications of such GMMs need to be addressed. Firstly, an appropriate design for the sustainability analysis of GMM applications needs to be provided by defining the aspects which should be addressed. Secondly, appropriate tests and the reference systems for comparison need to be chosen as well as well-defined system boundaries, which need to be based upon considerations for the specific GMM application in question. Also, an appropriate level and depth for the analysis has to be set by choosing an appropriate set of qualitative and quantitative case-specific indicators for the analysis.

For some issues, the data basis for a sustainability analysis of a specific issue is limited or lacking. Thus, further efforts are needed to address these knowledge gaps for the baseline situation as well as for the effects of the specific GMM applications.

The analysis provided in Section 4.1 for the two case studies of environmental applications of GMMs provides initial considerations on how to devise a sustainability analysis for certain GMMs. It also indicates some challenges that need to be addressed. An apparent example would be the choice of comparators, i.e., reference production systems, for applications such as GM microalgae for biofuel production, which will likely not be the one and only solution to achieving a transition to a renewable energy system. This raises the question which additional reference systems (ranging from plant-based biofuels or biogas and solar and wind energy) should be assessed in comparison. The example also illustrates the difficulties in addressing and accounting for indirect and long-term effects and how to deal with costs that are currently externalized to the public.

With regard to addressing societal concerns around the application of GMMs in the environment, TA approaches may be helpful for developing a more balanced governance approach to address ambiguous and quickly evolving socio-technological innovations [181,182]. Our survey of the current TA literature (see Section 4.2) suggests that the treatment of GMMs by TA is neither systematic nor comprehensive. The existing studies provide initial insights regarding potential societal and ethical issues with GMMs but cannot be considered a final opinion on their acceptability and the quality of the current governance. For the TA process, further efforts are necessary to address the socioeconomic issues outlined in Section 4.1. and to engage relevant stakeholders, including farmers, consumers, and environmental organizations to include their views on the application of GMMs.

## 6. Conclusions

The analysis of GM microalgae for biofuel production and GM soil bacteria for use as biofertilizers in cereal crop production, as examples for environmental applications of GMMs presented in this paper, indicates that a range of significant challenges is associated with both the risk assessment and the governance of such emerging applications. Our results suggest that the ERA of GMM applications is more challenging than the ERA of GM crop plants. This is mostly due to the current limitations in our understanding of microbial biology and ecology, which are a source for uncertainties relevant for the ERA of GMMs when released into the environment—either intentionally or unintentionally—as in the case of GM microalgae and GM biofertilizer agents. We stress that the existing guidance for risk assessment and monitoring of GMMs is insufficient to assess and mitigate associated risks for production systems and the environment. Therefore, we support the notion of EFSA [77,78] that the available guidance for GMMs needs to be updated, particularly for applications involving the environmental release of viable GMMs.

Based on the analysis of the case studies, we present recommendations to update the existing EU guidance regarding a number of issues, such as the microbial and molecular characterization of GMMs, the comparative assessment, the assessment of their potential toxicological, allergenic, and pathogenic characteristics, and a comprehensive assessment of the environmental risks of GMMs. The ERA of GMMs needs to comprise all areas of risks outlined by the EU biosafety legislation. Furthermore, specific guidance for the assessment of long-term exposure of the environment and for monitoring (e.g., PMEM) needs to be developed. Due to their highly fluctuating population dynamics, the risk assessment of microorganisms will benefit from modelling approaches, e.g., in silico models for simulations of GM microalgal biomass production under different environmental release scenarios and conditions. Furthermore, a comparative risk assessment, which is based on the comparison with non-GM taxa for which limited knowledge is available cannot be regarded as robust or conclusive. This necessitates the development of new assessment approaches for microorganisms, which lack a history of safe use. In general, further research efforts are needed to expand the available biosafety information, specifically when the existing knowledge on a particular parental microorganism is insufficient.

We conclude that GMM applications pose a number of significant challenges for their regulatory evaluation, specifically regarding the approaches for ERA, the assessment of long-term effects, the assessment of the impacts of consortia of different GMMs, and the monitoring of GMMs. For all of these aspects, appropriate methodologies need to be developed. In addition, we note that some pillars of the EU governance for GMOs, namely the time-limited authorization of GMO applications, is not feasible for GMMs, which do not naturally disappear, but can persist longer than 10 years in the environment after their initial release and which are not retrievable once released.

Substantial challenges also exist regarding the analysis of the sustainability of environmental applications of GMMs. According to our analysis, the sustainability analysis and the TA of GMM applications are less well developed than such approaches for GM crops. There is no agreed-upon practical assessment framework available for GMM applications and there are only limited data to address relevant issues, including socioeconomic and ethical issues. The further development and implementation of approaches for an evidence-based sustainability analysis of GMMs is thus necessary and may be based on the initial considerations regarding the design and framing of such an analysis presented in this article. In addition, the use of TA approaches to address wider societal concerns and the views of stakeholders regarding the use of GMMs in the environment should be considered. Such approaches would increase our ability to provide conclusive and robust advice to regulators concerning the acceptability of GMM applications, which involve release of the GMMs into the environment.

## Figures and Tables

**Table 1:** Examples for EU protection goals relevant for the RA of GMMs.

Protection Goal	Relevant Legislation/ Strategies	Objective	Relevant for RA of CS *
Soil quality and soil health	EU Soil Strategy for 2030 (COM(2021)699)	Protection and restoration of soil quality, health and biodiversity via a sustainable management	CS 1; CS 2
	Proposal for a Directive on Soil Monitoring and Resilience (COM(2023)416)	Establish a coherent soil monitoring framework for all soils across the EU to improve soil health	CS 1; CS 2
Water quality	EU Water Framework Directive (Directive 2000/62/EC)	Improvement of the ecological and chemical status of surface waters	CS 1; CS 2 (run-off water)
	EU Nitrate Directive 91/676/EEC	Minimizing nitrate input from agricultural sources into water bodies	CS 2
Ecosystem functions and services	Concept outlined by EFSA Scientific Committee [82]	Protection of relevant supporting, regulating, provisioning, and cultural services	CS 1; CS 2
Nature conservation	EU FFH Directive 92/43/EWG; Regulation (EU) 2024/1991 (Nature restoration law)	Protection and conservation of natural habitats and of wild animals and plants to restore, conserve, and promote biodiversity in natural habitats and to restore degraded terrestrial and aquatic ecosystems	CS 1; CS 2

* RA: risk assessment; CS: case study; CS 1: GM microalgae for biofuel production; CS 2: GM bacteria for use as biofertilizers.

**Table 2:** Risk issues for GM microalgae for biofuel production according to Directive 2001/18/EC and EFSA guidance [76,77].

Risk Issue ^1^	Area of Risk	Relevance for GM Microalgae (Risk Hypothesis Is Possible)
1/2	Survival/persistence/proliferation including selective advantage	Microalgae occur in natural habitats; ERA needs to address whether the GM trait provides a selective advantage (e.g., by affecting other microalgae or trophic levels)
3	Gene transfer (horizontal/vertical)	Relevant for sexually (e.g., *Chlamydomonas*) and asexually (e.g., *Nannochloropsis*, *Chlamydomonas*) reproducing taxa. Horizontal gene transfer to other algae/microorganisms is possible
5	Impacts on the biotic environment (e.g., non-target organisms)	Toxicological and nutritional effects of the specific changes in lipid composition on other organisms at the same or other trophic levels are possible
6	Interactions with the abiotic environment	Effects of the specific changes in lipid profile of GM microalgae on nutrient availability are possible
7	Environmental impacts of the specific techniques used for management of GMM	Relevant in case additional biocontainment traits (genetic or biological) would have adverse environmental impacts
8	Impacts on human and animal health	Toxicological and nutritional effects of changed levels of certain fatty acids are possible

^1^ according to the structure provided in Directive 2001/18/EC and [76].

**Table 3:** Risk issues for GMMs used as biofertilizers according to Directive 2001/18/EC and EFSA guidance [76,77].

Risk Issue ^1^	Area of Risk	Relevance for GM Microalgae (Risk Hypothesis Is Possible)
1/2	Survival/persistence/proliferation including selective advantage	Microalgae occur in natural habitats; ERA needs to address whether the GM trait provides a selective advantage (e.g., by affecting other microalgae or trophic levels)
3	Gene transfer (horizontal/vertical)	Relevant for sexually (e.g., *Chlamydomonas*) and asexually (e.g., *Nannochloropsis*, *Chlamydomonas*) reproducing taxa. HGT to other algae/microorganisms is possible
4	Impacts on the biotic environment (target organisms)	Potential plant-pathogenic effects under environmental conditions favoring colonization of certain plant species
5	Impacts on the biotic environment (non-target organisms)	Effects due to changes in the soil microbiome of the treated crop plots or unintended environments due to transport and spread of the GMMs. Such effects can be due to competition or the increased levels of bioavailable nutrients or nitrogen-compounds
6	Interactions with the abiotic environment	Effects on nutrient availability due to the nitrogen-fixation ability and other effects related to nutrient cycling in the soil (phosphate/sulfur solubilization, ability to increase the bioavailability of micronutrients)
7	Environmental impacts of the specific techniques used for management of GMM	Relevant depending on the specific use of the GMMs and the methods used to introduce the GMMs into the intended receiving environments (i.e., the treated soil for crop cultivation) Effects also depend on additional plant management interventions, e.g., further use of synthetic nitrogen fertilizers
8	Impacts on human and animal health	Increased virulence and pathogenicity may be possible Effects of exposure of the gut microbiome needs consideration

^1^ according to the structure provided in Directive 2001/18/EC and [76].

**Table 4:** Production systems which could be used for a sustainability analysis of GMM applications such as the use of microalgae for biofuels (CS 1) and microbial biofertilizers (CS 2) (PS: production system; TPS: test production system with GMMs, RPS: reference production system; N: nitrogen).

PS	CS 1 (Microalgae for Biofuels)	CS 2 (Microbial Biofertilizers in Cereal Production)
TPS1	Biofuel production with GM microalgae in open or semi-open pond systems	Supplementary use of GM biofertilizers (standard N fertilization plus GM biofertilizer)
TPS2	Biofuel production with GM microalgae in fully contained, closed-culture systems	Substitution of N fertilizer with GM biofertilizers (N fertilizer partly substituted with GM biofertilizer— minus 7.2 kg N (urea)/ha maize)
RPS1	Biofuel production with non-GM microalgae (in open or closed culture systems)	Biofertilizer use without additional N fertilizer (use of non-GM or GM biofertilizer)
RPS2	Production of standard fossil fuels	Synthetic and organic nitrogen fertilizers (standard use of synthetic/organic N fertilizers)

**Table 5:** Sustainability dimensions and themes for a sustainability analysis according to the SAFA guidelines, as well as relevant subthemes for both case studies. (GHG: greenhouse gas).

Sustainability Dimension *	Sustainability Themes *	GM Microalgae for Biofuel Production	GM Microbial Biofertilizers in Cereal Production
Ecological integrity	Atmosphere	GHG emissions, air pollutants	GHG emissions, air pollutants
	Water	Water Withdrawal, water Quality, waste water	Water Quality
	Soil	Land Use	Land Degradation
	Biodiversity	Ecosystem diversity, species diversity, genetic diversity	Ecosystem diversity, species diversity, genetic diversity
	Material and Energy	Energy used in production	Energy used in production
Economic resilience and efficiency	Production efficiency	Volume of production	Volume of production
	Economic resilience	Costs, energy security, affordability, and independence	Costs, affordability, and independence
Social sustainability	Decent Livelihood	Job creation, income	Farm income
	Equality, non-discrimination, gender equality, vulnerable groups	Human mobility	Opportunities for low-income and subsistence farmers
	Good governance	Compliance with energy and climate policies	Compliance with climate policies

* According to FAO [156].

## Supplementary Materials

The following supporting information can be downloaded at: https://www.mdpi.com/article/10.3390/ijms26073174/s1. References [81,82,129,183,184] are cited in Supplementary Materials.
