# Supplementary material for: Environmental Applications of GM Microorganisms: Tiny Critters Posing Huge Challenges for Risk Assessment and Governance
Source: Int J Mol Sci. 2025 Mar 29;26(7):3174. doi: 10.3390/ijms26073174 (PMC11989004; doi:10.3390/ijms26073174)
Supplement: Supplementary file 1 [file ijms-26-03174-s001.zip › ijms-3371370-supplementary.pdf]

Supplementary information S1: EU protection goals relevant for the risk assessment of GMMs, such as GM bacteria used as biofertilizer and GM microalgae for production of renewable fuels

1. EU Protection goals to be considered during ERA

The approach for ERA of GMOs as implemented in the EU is devised with a view to the protection goals laid out by the EU legislation or the respective national legislation [1]. EFSA has published guidance concerning specific protection goals for ERA, including protection goals relating to biodiversity and ecosystem services provided in the receiving environments which are exposed to a GMO [2]. In our review paper we provide examples for EU environmental protection goals which are relevant for the analysed case study GMMs (see Table 1 in chapter 3.2 of the main text). These protection goals are further discussed in the following sections.

1.1. EU protection goals for soil quality and soil health

The objectives addressed by the EU Soil Strategy for 2030 (COM(2021) 699) are of direct relevance for the risk assessment of GMMs used as biofertilizers as well as for unintended releases of GM microalgae that can affect soil quality, soil health and biodiversity as well as carbon and nutrient cycling.

The goals set by the EU soil strategy are interconnected with the targets of the EU Water Framework Directive, e.g. for water bodies impacted by agricultural management and fertilizer inputs. For the implementation of the EU Soil Strategy and to ensure that soils receive the same level of protection as water, air or the marine environment, a proposal for an EU Directive (COM(2023)416) is currently discussed, which should facilitate monitoring, protection, sustainable management and restoration of EU soils.

1.2. EU protection goals for water quality and biodiversity

The EU Water Framework Directive (Directive 2000/62/EC) and its targets to establish a good ecological and chemical status of surface waters in the EU until 2027 are relevant for both case study GMMs, and in particular for GM microalgae cultivation with a potential for spillage of the GMM and environmental exposure. The Directive aims to achieve a systematic improvement of freshwater habitats and avoid further deterioration of all waters in the EU. For classification of the ecological status, biotic quality components for waters are assessed, which comprise phytoplankton and phytobenthos, including microalgae. In this context, the frequency and intensity of algal blooms is considered an indicator of the good ecological status of water bodies. Directive 2000/62/EC is also relevant for GMMs from biofertilizers, which are deliberately released into agricultural soils and may be transported from the treated plots into freshwater habitats with run-off water. Additionally, the goals of reducing the nitrate input from agricultural sources into water bodies set by EU Nitrate Directive 91/676/EEC are relevant for GMM biofertilizers.

1.3. EU protection goals for ecosystem functions and services

Based on the concept of ecosystem functions and services outlined by the EFSA Scientific Committee [2], specific protection goals can be derived for the problem formulation of the ERA of GMOs, including GMMs.

Microalgae are key organisms at the basis of trophic food webs in many ecosystems and support important ecosystem functions and services. Microorganisms are components of phytoplankton [3] or of the microphytbenthos [4]. In many ecosystems, functions and the efficiency of services to mankind is linked to the microbial or microalgal biodiversity [5]:

- Supporting services: primary production, biogeochemical and nutrient cycling, sediment formation, protection from erosion, habitat formation;
- Regulating services: climate regulation, biological control and avoiding toxic algal blooms (microalgae), and eutrophication of water bodies;
- Provisioning services: food and drinking water, e.g. through commercial and recreational fisheries and water quality (microalgae), or by modulating health and productivity of certain crop plants [57] (soil bacteria);
- Cultural services: support of tourism and education (microalgae).

1.2. EU protection goals for nature conservation

The EU Fauna-Flora Habitat (FFH) Directive (92/43/EWG) and the more recent Regulation (EU) 2024/1991 (Nature restoration law) require to protect and conserve natural habitats (“Natura2000-sites”) and wild animals and plants, as well as to restore degraded terrestrial and aquatic ecosystems in the EU member states. Their targets are relevant for both case study GMMs, as microorganisms play important roles in the protected FFH sites and other ecosystems and are key components for conservation and ecosystem restoration efforts.

References

1. Devos, Y.; Aguilera, J.; Diveki, Z.; Gomes, A.; Liu, Y.; Paoletti, C.; Du Jardin, P.; Herman, L.; Perry, J.N.; Waigmann, E. EFSA's scientific activities and achievements on the risk assessment of genetically modified organisms (GMOs) during its first decade of existence: looking back and ahead. Transgenic Res 2014, 23, 1–25, doi:10.1007/s11248-013-9741-4.
2. EFSA Scientific Committee. Guidance to develop specific protection goals options for environmental risk assessment at EFSA, in relation to biodiversity and ecosystem services. EFSA Journal 2016, 14, doi:10.2903/j.efsa.2016.4499.
3. Naselli-Flores, L.; Padisák, J. Ecosystem services provided by marine and freshwater phytoplankton. Hydrobiologia 2023, 850, 2691–2706, doi:10.1007/s10750-022-04795-y.
4. Hope, J.A.; Paterson, D.M.; Thrush, S.F. The role of microphytobenthos in soft-sediment ecological networks and their contribution to the delivery of multiple ecosystem services. Journal of Ecology 2020, 108, 815–830, doi:10.1111/1365-2745.13322.
5. Zhou, S.; Li, W.; He, S. Microalgal diversity enhances water purification efficiency in experimental microcosms. Front. Ecol. Evol. 2023, 11, doi:10.3389/fevo.2023.1125743.
